# Alteration of Metabolic Profile in Patients with Narcolepsy Type 1

**DOI:** 10.3390/metabo15060382

**Published:** 2025-06-09

**Authors:** Md Abdul Hakim, Waziha Purba, Akeem Sanni, Md Mostofa Al Amin Bhuiyan, Farid Talih, Giuseppe Lanza, Firas Kobeissy, Giuseppe Plazzi, Fabio Pizza, Raffaele Ferri, Yehia Mechref

**Affiliations:** 1 Department of Chemistry and Biochemistry, Texas Tech University, Lubbock, TX 79409, USA; md-abdul.hakim@ttu.edu (M.A.H.); wpurba@ttu.edu (W.P.); aksanni@ttu.edu (A.S.); mdmobhui@ttu.edu (M.M.A.A.B.); 2 Department of Psychiatry, Faculty of Medicine, American University of Beirut, Beirut 1107 2020, Lebanon; ft10@aub.edu.lb; 3 Sleep Research Centre, Department of Neurology IC, Oasi Research Institute-IRCCS, 94018 Troina, Italy or giuseppe.lanza1@unict.it (G.L.); rferri@oasi.en.it (R.F.); 4 Department of Surgery and Medical-Surgical Specialties, University of Catania, 95123 Catania, Italy; 5 Department of Biochemistry and Molecular Genetics, Faculty of Medicine, American University of Beirut, Beirut 1107 2020, Lebanon; firasko@gmail.com; 6 Department of Neurobiology, Center for Neurotrauma, Multiomics and Biomarkers (CNMB), Neuroscience Institute, Morehouse School of Medicine (MSM), Atlanta, GA 30310-1458, USA; 7 IRCCS, Instituto delle Scienze Neurologiche di Bologna, 40139 Bologna, Italy; giuseppe.plazzi@unibo.it (G.P.); fabio.pizza@unibo.it (F.P.); 8 Department of Biomedical, Metabolic and Neural Sciences, University of Modena and Reggio Emilia, 41121 Modena, Italy; 9 Department of Biomedical and Neuromotor Sciences (DIBINEM), Alma Mater Studiorum, University of Bologna, 40123 Bologna, Italy

**Keywords:** autoimmunity, hypocretin, cataplexy, biomarker, neuronal cell death

## Abstract

**Background:** Narcolepsy type 1 (NT1) is a rare neurological sleep disorder characterized by excessive daytime sleepiness and cataplexy. NT1 is thought to be caused by the loss of hypocretin-producing neurons in the hypothalamus due to autoimmunity. Since cerebrospinal fluid hypocretin testing is invasive and not always feasible in clinical practice, there is a critical need for less invasive biomarkers to improve diagnostic accuracy and accessibility. Very few studies have explored serum-based biomolecules that could serve as biomarkers for NT1. **Methods:** This study examines the differential abundance of serum metabolites in patients with NT1 using an LC-MS/MS-based comprehensive metabolomics approach. **Results:** An untargeted analysis identified a total of 1491 metabolites, 453 of which were differentially abundant compared to the control cohort. Ingenuity pathway analysis revealed that key pathways, such as the inflammatory response (*p*-value of 0.01, activation z-score of 0.5), generation and synthesis of reactive oxygen species (*p*-value of 0.0008, z-score of 1.3), and neuronal cell death (*p*-value of 0.04, z-score of 0.4), are predicted to be activated in NT1. A targeted analysis using parallel reaction monitoring validated 49 metabolites, including important downregulated metabolites such as uridine (fold change (FC) of 0.004), epinephrine (FC of 0.05), colchicine (FC of 0.2), corticosterone (FC of 0.3), and arginine (FC of 0.6), as well as upregulated metabolites such as p-cresol sulfate (FC of 2601.7), taurine (FC of 1315.4), inosine (FC of 429.7), and malic acid (FC of 7.9). **Conclusions:** Understanding the pathways identified in this study and further investigating the differentially abundant metabolites associated with them may pave the way for gaining insight into disease pathogenesis and developing novel therapeutic interventions.

## 1. Introduction

Narcolepsy is a severe neurological disorder accompanied by excessive daytime sleepiness (EDS) and emotion-driven loss of muscle tone, known as cataplexy [1,2,3,4]. People with narcolepsy often experience disturbed sleep, hypnagogic hallucinations, and sleep paralysis [5,6]. People affected with narcolepsy worldwide are approximately 25–50 per 100,000 individuals [7]. The frequent misdiagnosis hampers the initiation of proper treatment. Narcolepsy type 1 (NT1) is distinguished from narcolepsy type 2 in the presence of cataplexy and a lower extent of cerebrospinal fluid (CSF) hypocretin (also known as orexin), a neuropeptide involved in the regulation of the sleep–wake cycle [8,9,10,11]. The interaction between hypocretin-producing neurons in the lateral hypothalamus and noradrenergic neurons in the locus coeruleus is involved in the hypocretin-mediated regulation of the sleep–wake cycle [12]. While hypocretin neurons become silent during sleep, they are highly active when an individual is awake. These neurons exhibit bursts of activity that help trigger the transition from sleep to wakefulness [13]. Through orexin receptors, hypocretin promotes the activation of monoaminergic and cholinergic systems, which facilitate cortical activity and sustain wakefulness [14]. In NT1, autoimmune processes are believed to cause the selective loss of hypocretin-producing neurons in the lateral hypothalamus [15]. The severity of NT1 is elevated by genetic susceptibility, particularly the human leukocyte antigen (HLA) DQB1*06:02 allele, a genetic variant of the HLA-DQB1 gene that plays critical roles in the immune system [4,16]. The HLA-DQB1*06:02 allele has been strongly associated with NT1, and studies have reported that about 90% of NT1 patients carried this gene and had low levels of CSF hypocretin [16,17,18]. Besides regulating the sleep–wake state, hypocretin is also involved in the regulation of appetite and energy production metabolism [19]. Sleep cycle imbalance and metabolic imbalance are observed due to the inhibition of hypocretin signaling in NT1 patients [20]. Due to the strong association of the (HLA) DQB1*06:02 allele and hypocretin deficiency, clinicians often suggest HLA typing first prior to sending the patients for more invasive CSF analysis to diagnose NT1 [12].

Metabolites refer to small molecules (typically less than 1500 Da) that play important roles in biological systems by contributing to cellular and physiological processes, including forming cell membranes, activating nuclear receptors, regulating signaling transduction, and producing energy [21]. Unlike protein and RNA, which result from the transcription and translation of unique genome codes, metabolites are the intermediate or end products of cells’ different biological, biochemical, and metabolic pathways [22]. Therefore, studying metabolomes through the complete characterization and quantification of all metabolites in a cell, tissue, or organism provides information about the altered expression of certain metabolites and metabolic pathways, enabling us to find their roles in complex biological pathways and their association with pathogenesis [23,24]. As a result, metabolomics has become a potential area of biomarker research, facilitating the early diagnosis of disease and monitoring disease progression and the effectiveness of treatment [25,26,27]. Though the loss of hypocretin-producing neurons is an established characteristic sign of NT1, metabolomic studies can provide insights into much broader biochemical alterations associated with NT1, as reported for other sleep disorders [28]. Beyond genetic markers, such as HLA-DQB1*06:02, and hypocretin deficiency, metabolomic analysis offers a more dynamic and systems-level understanding of NT1 pathophysiology [29]. Recent studies have shown significant alterations in metabolites, including amino acid, lipid, and energy metabolism, as well as disruptions in metabolic pathways such as glycine and serine, arachidonic acid, and tryptophan metabolism in NT1 [29,30,31]. These findings reinforce the significance of metabolomics in the search for potential diagnostic biomarkers and therapeutic targets and in enhancing our understanding of NT1 pathogenesis beyond genetic predisposition and hypocretin loss.

The current challenges of metabolomics include the lack of a high-throughput analytical method to analyze a vast number of samples, the detection of metabolites from the injection of minute amounts of samples, and proper validation techniques to confirm the identification and level of expression in the system [32,33]. Liquid chromatography–tandem mass spectrometry (LC-MS/MS) is a powerful technique used in proteomics, glycoproteomics, glycomics, lipidomics, and metabolomics [34,35,36,37,38,39]. It enables the simultaneous separation, detection, and quantification of thousands of metabolites with high sensitivity and selectivity. The samples can be analyzed in negative and positive ionization modes, increasing metabolite coverage. Moreover, targeted experiments such as parallel reaction monitoring can be used to validate the identified metabolites in the samples. With the help of bioinformatics tools, the raw files with large datasets can be converted into biologically meaningful results [40].

LC-MS/MS-based metabolomics is a widely used approach to study the molecular mechanisms of various human diseases, such as cardiac, kidney, and neurodegenerative diseases, including heart failure, myocardial infarction, coronary artery disease, chronic kidney disease, acute kidney injury, diabetic nephropathy, polycystic kidney disease, Alzheimer’s disease, Parkinson’s disease, and amyotrophic lateral sclerosis, and discover novel biomarkers [41,42,43]. Among the neurodegenerative diseases, metabolomics showed promise in tracking pathogenic pathways and mechanisms during the progression of Alzheimer’s, Parkinson’s, and Huntington’s diseases [44,45,46,47,48]. The alteration of purine metabolites and the glycerophospholipid and linoleic pathways, for example, was observed in Parkinson’s disease [45,49]. Several studies utilized metabolomics approaches to reveal the correlation of altered metabolic profiles and metabolic pathways with sleep disorders and sleep insufficiency [28,50,51]. Despite having several metabolomics studies investigating the pathogenesis of different neurological, metabolic, and sleep disorders, there is limited research identifying and validating serum metabolite markers that can be confidently used for the early diagnosis of NT1 [29]. In this study, we investigated the alteration of serum metabolic profiles in patients with NT1 in search of candidate biomarkers. We also investigated important biological and metabolic pathways affected by the altered metabolites during NT1 progression. A targeted analysis, parallel reaction monitoring (PRM), was conducted to validate the differentially abundant metabolites and establish their potential to be considered biomarkers for NT1.

## 2. Materials and Methods

### 2.1. Chemicals and Reagents

HPLC-grade methanol, acetonitrile, water, dichloromethane (DCM), and formic acid were purchased from Fisher Scientific (Fair Lawn, NJ, USA).

### 2.2. Study Participants

A total of 11 NT1 patients and 11 healthy people participated in this study. A written consent was obtained from each participant before conducting the study. The study was also approved by the local ethics committee. The blood sera were collected from patients who were clinically diagnosed with NT1 and were admitted to the Department of Neurology and Sleep Medicine, Oasi Research Institute, Troina, Italy, and the Department of Neurology, University of Bologna, Italy. Patients with clinical findings such as diabetes, high blood pressure, obesity, and other neurodegenerative complications apart from NT1 were not included in this study. To reduce potential confounding factors, we excluded individuals with significant comorbidities such as diabetes, hypertension, obesity, or neurodegenerative disorders that could independently affect sleep architecture, autonomic regulation, or motor activity during sleep. This approach was chosen to ensure that the observed findings could be more confidently attributed to NT1 itself rather than to other overlapping conditions.

The presence of human leukocyte antigen (HLA) DQB1*0602, an antigenic peptide that plays a role in the immune system, was tested. The patients had no previous record of having a *Streptococcus* infection or H1N1 flu, nor did they take the AS03-adjuvanted vaccine prior to growing symptoms of narcolepsy. The patients were not taking any medications before and during the blood sampling. The samples were collected between 9 a.m. and noon over the period from April 2018 to June 2018. In sleep medicine, it is considered good practice to assess biological processes during a consistent chronobiological window, given that sleep and its associated physiological markers are tightly regulated by circadian rhythms. For this reason, all recordings and sample collections were performed between 9 a.m. and 12 p.m. to minimize variability due to circadian influences and to ensure comparability across subjects. This time frame was selected to capture the post-sleep period while maintaining consistency within the same circadian phase.

Healthy control subjects were recruited through institutional advertisements and underwent a semi-structured clinical interview to confirm the absence of sleep complaints, neurological or psychiatric disorders, and any regular medication use; all reported a stable lifestyle, regular sleep–wake schedules, no habitual daytime sleep, and good subjective sleep quality. Some clinical information for NT1 patients and healthy controls is shown in Table 1.

For all NT1 patients, a full-night polysomnographic recording was available as part of their diagnostic workup in the sleep laboratory. Patients were allowed to sleep until a spontaneous morning awakening. The following signals were recorded: electroencephalogram (at least three channels: one frontal, one central, and one occipital, each referred to the contralateral earlobe); electrooculogram (two channels); electromyogram of the submentalis muscle and both tibialis anterior muscles; electrocardiogram (single lead); and standard respiratory channels, including airflow, respiratory effort, peripheral oxygen saturation, and body position. Sleep stages were visually scored in 30 s epochs according to standard criteria. Table 2 presents the main polysomnographic features of the NT1 patients, which are consistent with typical findings for this population.

### 2.3. Metabolite Extraction

**Figure 1:**
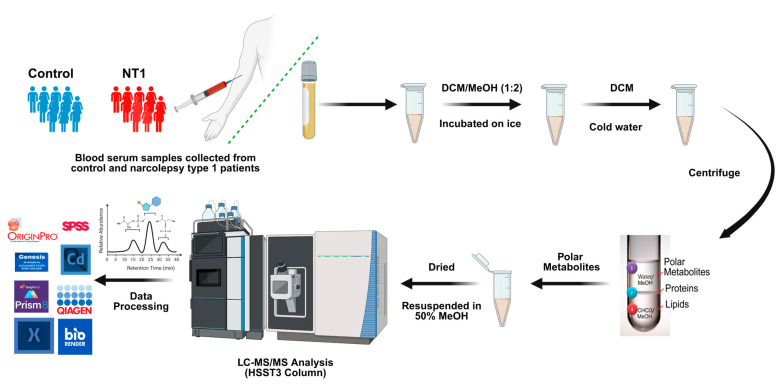
Workflow of metabolomics study summarizes the collection of samples from patients, sample preparation, extraction of the polar metabolites, analyzing them in LC-MS/MS, and data processing.

The metabolite extraction was developed from the previously established methods published in the literature [52,53]. Briefly, an aliquot of 100 µL serum of each sample was transferred to a 1.5 mL Eppendorf tube, followed by the addition of 200 µL of solvent mixtures prepared with dichloromethane and methanol at a 1:2 (*v/v*) ratio. Uniform mixing was ensured by vortexing the samples for 30 s. The samples were then incubated for one hour at an ambient temperature. An additional 75 µL of DCM was added to the samples, followed by vortexing for 30 s. The mixture was vortexed again after adding 75 µL of cold water to the samples. At the end, to separate and collect the metabolites, the samples were centrifuged at 5000 rpm for 15 min, which transformed the mixture into three distinct layers containing metabolites, proteins, and lipids. The upper layer is aqueous, and it contains metabolites. The upper layer was carefully transferred to another Eppendorf tube and then dried and resuspended with a solution of 50% methanol and 50% water. A brief workflow of this study is illustrated in Figure 1.

### 2.4. LC-MS/MS Parameters

A Vanquish UHPLC system (Thermo Scientific, San Jose, CA, USA) interfaced with a Q Exactive HF was used to analyze the extracted metabolites. The separation was performed on an Acquity UPLC HSST3 100 Å (2.1 × 100 mm) column (Waters, Wexford, Ireland) using a multistep gradient. Water containing 0.1% FA and methanol with 0.1% FA were used as mobile phase A (MPA) and mobile phase B (MPB), respectively, and the flow rate was set to 0.4 µL/min.

The elution gradient started with 0.5% MPB and gradually increased to 50% over the first 5.5 min. The MPB was then ramped to 98% over 30 s and maintained at 98% for 6 min. From 12–13 min, the MPB was decreased to the initial condition of the run and kept constant for the last 2 min to equilibrate the column. The separated metabolites were sent to the mass spectrometer via an analytical ESI source. The samples were analyzed in both positive and negative ionization modes, and spectra were obtained in the data-dependent acquisition mode. The spray voltage was set to 3.5 kV while running samples in positive mode and 3.0 kV in negative mode. The temperatures of the transfer tube were 300 °C and 320 °C in positive and negative ionization modes, respectively. The full MS spectra were acquired with a scan range of 75–750 *m/z*, the resolution was set to 120 K, the AGC target was 3 × 10^6^, and the maximum injection time was set to 200 ms. To get rid of unexpected ions coming from the solvent matrix, an exclusion list prepared with the top 100 most intense peaks from the first 6 min of the blank run was included in the instrument method. The top 4 most intense ions were subjected to an MS2 event with a scan range of 75–2000 *m/z*. The higher-energy collision dissociation (HCD) fragmentation was used with a stepped normalized collision energy of 20%, 40%, and 60%. The isolation window was 4 *m/z*, and dynamic exclusion was 6 s in positive mode, whereas the isolation window was 1 *m/z*, and dynamic exclusion was 6 s again. The resolution was set to 30,000 in positive mode and 15,000 in negative mode, and the maximum injection time was set to 50 ms.

### 2.5. Data Analysis

The raw files were processed in Compound Discoverer 3.1. The Mann–Whitney *U* test was performed on all identified metabolites with Benjamini–Hochberg correction. An unsupervised principal component analysis was performed on Origin Pro 2.0 to show the clustering of disease samples compared to the control. A heatmap of significant metabolites was plotted using Genesis 1.8.1. Box plots of the important metabolites were created with the relative abundance of the metabolites in GraphPad Prism 10.0.2.

### 2.6. Ingenuity Pathway Analysis

Ingenuity pathway analysis (IPA) was performed on statistically significant metabolites to determine the roles of different metabolites in modulating biological pathways, bio-functions, and diseases with the use of QIAGEN IPA software, version 134816949. To inform the metabolites in the software, identification numbers from the Human Metabolome Database (HMDB), PubChem, and the Kyoto Encyclopedia of Genes and Genomes (KEGG) were included. To start the analysis, the logarithmic fold change (log2FC) values and *p*-values of all differentially abundant metabolites were imported into the software. IPA used *p*-values and z-scores to group the metabolites into canonical pathways. Moreover, the software identified associations between biological functions, diseases, and metabolites by mapping their interactions and correlations. While *p*-value (<0.05) indicates the statistical significance of each cluster, z-score is used to predict the activation (positive z-score) or inhibition (negative z-score) of upstream regulators and downstream functions, as well as pathways [54].

### 2.7. Validation with LC-PRM-MS

Significant metabolites with high levels of fold changes that are found to be involved in important biological pathways in ingenuity pathway analysis were selected for targeted analysis for further validation. Xcalibur software (Thermo Scientific) version 4.4.16.14 was used to manually check the chromatographic peaks of the selected metabolites and confirm their retention time from a pooled sample analyzed on the instrument. With the metabolite name, structural formula, correct retention time, and *m/z* value, the transition list of metabolites was prepared for the targeted experiment. A total of 130 metabolites were targeted. For PRM analysis, the same LC gradient and mass spectrometry parameters were used as in the untargeted experiment. The raw files of PRM analysis were processed in Skyline software version 23.1.0.380, and statistical analysis was applied to the relative abundance of metabolites.

## 3. Results

### 3.1. Unsupervised Principal Component Analysis of All Metabolites

**Figure 2:**
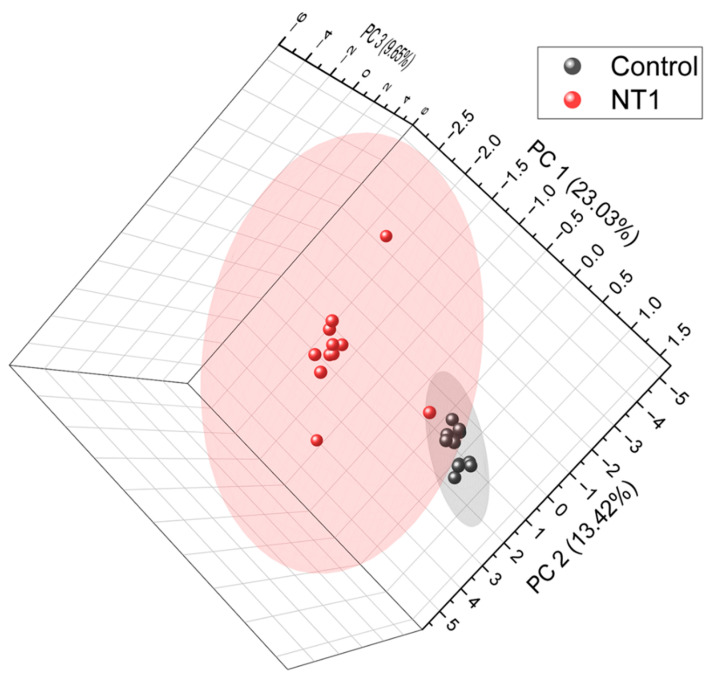
Unsupervised principal component analysis (PCA) of all metabolites identified from NT1 and control samples. PCA was plotted at a 95% confidence level.

A total of 1491 metabolites were identified and quantified. The relative abundance of all the metabolites was used to plot unsupervised principal component analysis (Figure 2) in order to see the clustering of two cohorts and to assess whether the metabolites can be a factor in distinguishing the diseased individuals from healthy controls. From the PCA, we can see a clear separation between the two cohorts. The control samples formed a separate cluster, which is distinct from the cluster of NT1 samples. This indicates that metabolites can be a key factor in differentiating NT1 from healthy controls. A list of all differentially abundant metabolites, average relative abundance in control and NT1 samples, *p*-value, fold change, and log2 of fold change is included in Supplementary Table S1.

### 3.2. Heatmap of Differentially Abundant Metabolites

**Figure 3:**
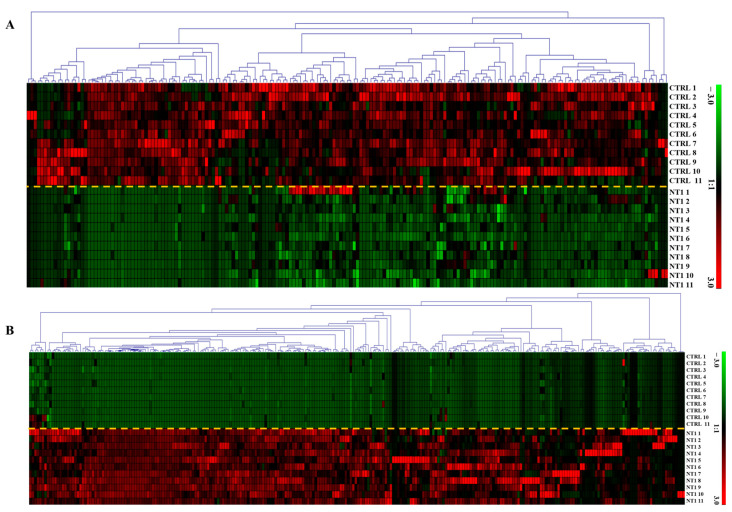
Heatmap of differentially abundant (**A**) downregulated and (**B**) upregulated metabolites in NT1 patients compared to control. Rectangular boxes with red and green colors represent the up- and downregulation of the metabolites, respectively The yellow dashed line is used to separate the two cohorts The *y*-axis shows an expression range from +3 to −3, where red at +3 represents the highest level of upregulation, and green at −3 indicates the highest level of downregulation.

Out of 1491 identified and quantified metabolites, 453 were found to be statistically significant (*p*-value < 0.05). We found 262 metabolites upregulated and 191 metabolites downregulated. Figure 3A shows the expression of 191 metabolites found downregulated, and Figure 3B visualizes the upregulation of 262 metabolites in NT1 compared to the control group.

### 3.3. Ingenuity Pathway Analysis

**Figure 4:**
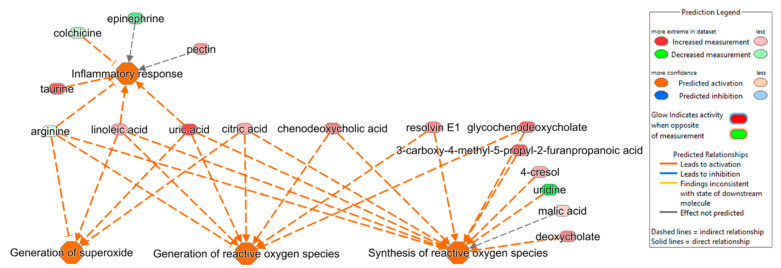
Ingenuity pathway analysis shows the correlation of DAMs in activating pathways such as inflammatory response (*p*-value of 0.01, z-score of 0.5), generation of superoxide (*p*-value of 0.001, z-score of 2.0), generation of reactive oxygen species (ROS) (*p*-value of 0.0008, z-score of 1.3), and synthesis of ROS (*p*-value of 0.001, z-score of 1.3).

Differentially abundant metabolites (DAMs) were used to perform an ingenuity pathway analysis to investigate key biological pathways that are involved in NT1 progression. Several metabolites, either upregulated or downregulated, were found to be associated with important biological pathways such as inflammatory response, generation of reactive oxygen species (ROS), and generation of superoxide (Figure 4). The inflammatory response is predicted to be activated due to the upregulation of metabolites such as taurine, pectin, linoleic acid, and uric acid, and the downregulation of colchicine and arginine. Among the metabolites involved in the inflammatory response, arginine, linoleic acid, and uric acid were also found to be responsible for activating the pathways named generation of superoxide and generation of ROS. In addition, the upregulation of citric acid was found to be involved in both the generation of superoxide and ROS pathways. Resolvin E1, glycochenodeoxycholate, 3-carboxy-4-mehtyl-5-propyl-2-furanpropanoic acid, 4-cresol, uridine, malic acid, and deoxycholate were associated with the predicted activation of the synthesis of the ROS pathway.

**Figure 5:**
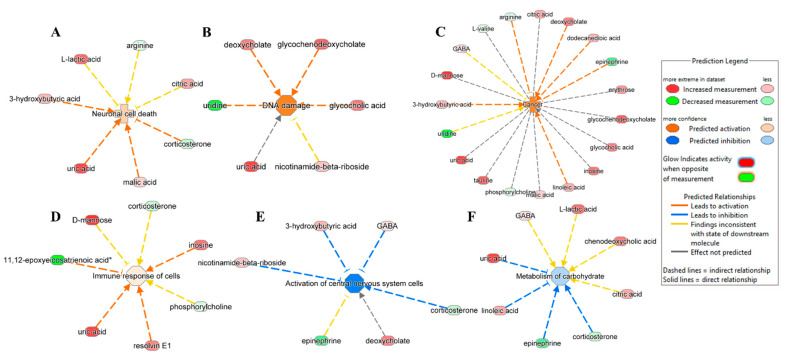
Ingenuity pathway analysis shows DAMs involved in the predicated activation of disease and function: (**A**) neuronal cell death (*p*-value of 0.04, z-score of 0.4), (**B**) DNA damage (*p*-value of 0.004, z-score of 1.4), (**C**) cancer (*p*-value of 0.000007, z-score of 1.3), and (**D**) immune response of cells (*p*-value of 0.03, z-score of 0.2). (**E**) Activation of the central nervous system (*p*-value of 0.0001, z-score of −1.4) and (**F**) metabolism of carbohydrates (*p*-value of 0.004, z-score of −0.4) were predicted to be inhibited in NT1.

While looking into the association of metabolites in disease and functions in NT1, IPA predicted the activation of neuronal cell death (Figure 5A), DNA damage (Figure 5B), cancer (Figure 5C), the immune response of cells (Figure 5D), the inhibition of central nervous system cells, and the activation (Figure 5E) and metabolism of carbohydrate (Figure 5F).

IPA predicted activation of neuronal cell death caused by the overexpression of 3-hydroxybutyric acid, uric acid, and malic acid and the underexpression of corticosterone. As for the activation of DNA damage, among the associated metabolites, deoxycholate, glycochenodeoxycholate, and glycocholic acid were upregulated, and uridine was downregulated. IPA also indicates the possibility of cancer emergence due to an altered expression of several metabolites such as arginine, deoxycholate, dodecanoic acid, epinephrine, linoleic acid, and 3-hydroxybutyric acid. We observed the upregulation of inosine, resolving E1, uric acid, and downregulation of 11,12-epoxyeicosatrienoic acid, leading to the activation of the immune response of cells. Two important pathways were predicted to be inhibited, including the activation of central nervous system cells and the metabolism of carbohydrates. The activation of central nervous system cells was hindered by the upregulated metabolites 3-hydroxybutyric acid, gamma-amino-butyric acid (GABA), deoxycholate, and nicotinamide-beta-riboside, and it downregulated corticosterone. The inhibition of the metabolism of carbohydrates was prompted by uric acid and linoleic acid being in an upregulated state and epinephrine and corticosterone being in a downregulated state.

While investigating disease networks from ingenuity pathway analysis, we found that differential expressions of some metabolites are responsible for activating or inhibiting pathways, including cell-to-cell interaction, free radical scavenging, and nervous system functions (Supplementary Figure S1). The association of the metabolites in several diseases and functions is also shown, while some metabolites are previously reported as potential biomarkers for Alzheimer’s and Parkinson’s disease. Another network shows the metabolites involved in activating pathways such as inflammatory response, organismal injury, and abnormalities. IPA also shows that those metabolites affect nervous system function, leading to neurodegeneration (Supplementary Figure S2).

### 3.4. Validation of DAMs by PRM

After the identification, quantification, and statistical analysis of the differentially abundant metabolites that show high levels of fold change and those that are involved in important biological pathways, a list of DAMs was prepared to conduct and validate their level of expression using targeted PRM analysis. We were able to validate 49 metabolites. These metabolites, validated with PRM, followed a similar trend of fold change as observed in the untargeted analysis. The list of validated metabolites, their *m/z* value, transition ion fragments, fold change, and log2 value of fold change of the full scan and PRM experiments are included in Supplementary Table S2.

### 3.5. Box Plots and ROC Analysis of DAMs Associated with Important Biological Pathways

After conducting an ingenuity pathway analysis, box plots were made for the validated DAMs that were found to be involved in important biological pathways, diseases, and functions (Supplementary Figures S3 and S4).

Supplementary Figure S3 shows the box plots of some upregulated DAMs, and Supplementary Figure S4 shows the box plots of some of the important DAMs that are found to be involved in implicating different biological pathways during ingenuity pathway analysis and discussed in the previous section. Among the upregulated DAMs, p-cresol sulfate, taurine, inosine, and malic acid showed the same *p*-value of 1.43 × 10^−5^ and with Log2 of fold change (Log2FC) values of 11.34, 10.36, 8.75, and 2.97, respectively. They all followed the same level of expression in PRM validation, while p-cresol sulfate remained statistically significant. Among the important downregulated DAMs, the box plots of uridine (*p*-value 1.43 × 10^−5^, Log2FC −7.81), epinephrine (*p*-value 0.002, Log2FC −4.46), colchicine (*p*-value 0.0002, Log2FC −02.21), corticosterone (*p*-value 1.43 × 10^−5^, Log2FC −2.00), and arginine (*p*-value 0.001, Log2FC −0.59) are shown in Supplementary Figure S4.

**Figure 6:**
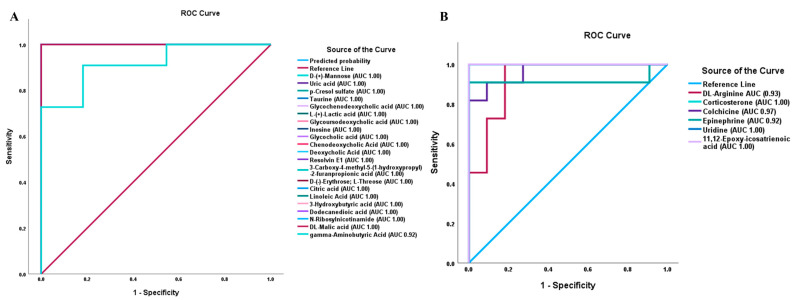
Receiver Operating Characteristics (ROC) curve and area under the curve (AUC) values of differentially abundant metabolites in NT1. (**A**) ROC curves and AUC values of important upregulated DAMs. (**B**) ROC curves and AUC values of important downregulated DAMs.

To evaluate how sensitive and specific the DAMs are in distinguishing NT1 from control, we plotted the Receiver Operating Characteristics (ROC) curve. We determined the area under the curve (AUC) value of differentially abundant metabolites. The closer the AUC value is to 1, the more it is a distinguishing factor. From the ROC analysis and AUC values, it was observed that most of the upregulated metabolites that are biologically relevant and associated with different important pathways maintain a perfect AUC score of 1.00. In Figure 6A, all metabolites showed an AUC score of 1.00 except gamma-aminobutyric acid, which also has a high AUC value (0.92). While plotting ROC curves of important downregulated DAMs, we observed AUC value ranges from 0.92 to 1.00, with arginine having an AUC value of 0.93, corticosterone 1.00, colchicine 0.97, epinephrine 0.92, uridine 1.00, and 11,12-epoxy-icosatrienoic acid 1.00 (Figure 6B).

## 4. Discussion

Narcolepsy type 1, a sleep disorder, is characterized by a decreased expression of hypocretin neurons in the hypothalamus [4]. This improper functioning of the hypocretinergic system impacts the metabolic pathways and often complicates existing metabolic conditions such as diabetes and metabolic syndrome [55]. Although cerebrospinal fluid hypocretin measurement is currently the gold standard for diagnosing NT1 [56], it requires a lumbar puncture, which is invasive and not routinely feasible in many clinical settings. Therefore, there is a pressing need for minimally invasive and widely accessible biomarkers, such as those detectable in blood, to improve diagnostic accuracy and facilitate broader clinical implementation. Previously, attempts have been made to identify and validate protein, glycan, and glycopeptide-based candidate markers to facilitate the correct diagnosis of NT1 at an early stage [34,57,58]. This work attempts to find metabolite markers of NT1 through a comprehensive investigation of differentially abundant metabolites from the serum of 11 NT1 patients compared to 11 healthy control samples. A total of 1,491 metabolites were identified and quantified, and 453 of them were differentially abundant. We looked into the molecular effects and cellular response due to the changes in the expression of metabolites by utilizing bioinformatics tools such as ingenuity pathway analysis (IPA).

The inflammatory response can be triggered by the activation of the acute phase signaling pathway to protect the body against any infection or damage from tissue injury. Acute phase signaling has previously been linked to the alteration of physiological systems, such as EDS, which is also very common in narcolepsy [59]. Increased levels of inflammatory markers and pro-inflammatory cytokines and decreased levels of anti-inflammatory cytokines have been reported in studies on patients with obstructive sleep apnea [60,61,62]. Sanni et al. reported several proteins involved in acute phase response signaling in patients with NT1 [34]. In this study, IPA results showed that an inflammatory response was predicted to be activated, which supports the previous findings. Increased levels of linoleic acid have previously been shown to be associated with inflammation and metabolic diseases [63,64]. High levels of uric acid were also reported to be associated with inflammatory markers and stimulate inflammation in cardiovascular disease patients [65]. Our results showed that both linoleic acid and uric acid were upregulated in NT1, which might be responsible for activating the inflammatory response in NT1 patients.

Reactive oxygen species (ROS) such as superoxide anion, hydrogen peroxide, and hydroxyl radical are involved in various important bio-functions such as cell signaling, metabolism, homeostasis, and formation of memory through the methylation of DNA [66,67]. However, the overexpression of ROS can lead to the development of neurodegenerative diseases [68]. Neurons consist of high polyunsaturated fatty acids, and their antioxidant defense mechanism is weak; therefore, the overproduction of ROS can cause the oxidative degradation of neurons [68,69]. The synthesis of ROS mainly takes place in the mitochondria during oxidative phosphorylation, and the excessive production of ROS can cause cellular damage by damaging the mitochondrial DNA [70]. Our study reveals that the generation of ROS was activated in NT1, and more interestingly, IPA predicted the activation of neuronal cell death and DNA damage, which are likely to happen because of the overproduction of ROS. Moreover, the activation of central nervous system cells was also prevented and was linked to some of the DAMs found in the study.

Metabolic disorders such as type-2 diabetes have previously been reported to be found in patients with narcolepsy who have hypocretin deficiency [71]. Our data shows that the metabolism of carbohydrates was predicted to be inhibited, indicating the progression of diabetes in NT1 patients. A study on the serum metabolomics of insomnia patients demonstrated metabolic dysregulation due to the inhibition of glucose metabolism and amino acid metabolism in insomnia patients compared to good sleepers, and they reported a number of metabolites, such as 3-hydroxybutyric acid, citric acid, and lactic acid, that were differentially abundant in insomnia patients. Our study also found these metabolites elevated and differentially abundant in NT1.

Among the downregulated DAMs, arginine, corticosterone, colchicine, epinephrine, uridine, and 11,12-epoxy-icosatrienoic acid are found to be involved in the predicted activation/inhibition of important biological pathways, diseases, and functions during NT1 progression. L-arginine is the precursor of the synthesis of nitric oxide (NO), an important chemical messenger and regulator involved in many physiological functions of the body, including the central nervous system (CNS) [72]. Maintaining optimal blood flow in the brain, consolidating the memory process, regulating the sleep–wake cycle, controlling appetite and temperature of the body, and playing a role in normal olfactory function are some of the important functions of NO [73,74]. The dysregulation of NO has been reported to be associated with nitroxidative stress and the development of neurodegenerative diseases such as Alzheimer’s disease and Parkinson’s disease [75]. As L-arginine is converted to NO in the presence of nitric oxide synthase enzyme, the dysregulation of L-arginine can directly alter the level of NO in the body and, therefore, negatively impact the normal function of the central nervous system. Decreased levels of serum L-arginine have been reported in cases of dementia patients [76]. Ahmed et al. reported a decreased level of arginine in plasma samples collected from patients with Parkinson’s disease [77]. In this study, downregulated arginine is seen to be associated with the activation of the inflammatory response, generation of ROS, and neuronal cell death.

Epinephrine, also known as adrenaline, is a neurotransmitter and hormone that is involved in the body’s fight-or-flight response at times of stress or danger. The dysregulation of important biochemicals, such as neurotransmitters or hormones, can pose a risk of developing different kinds of neurological disorders [78]. The downregulation of epinephrine has been reported to cause anxiety, depression, and alteration of blood pressure [79]. All these symptoms can exacerbate an individual’s normal sleep–wake regulation and might lead to NT1. An untargeted serum metabolomics study on young teenagers with NT1 showed significant downregulation of epinephrine, dodecanoic acid, and 2-heptanone in NT1 compared to healthy individuals [29]. A recent study on rats exposed to glyphosate-based herbicides showed that there is a reduced level of epinephrine found in the exposed rats, and their findings also suggested that this low level of epinephrine can be associated with developing neurophysiological disorders in the rats [80]. Interestingly, we also found a significant downward trend of epinephrine in NT1 patients, and IPA showed that this downregulation of epinephrine is involved in several diseases and functions, such as activation of cancer progression, inhibition of central nervous system cells, and inhibition of carbohydrate metabolism.

Uridine, a pyrimidine nucleoside, is found in the biological fluid at an elevated concentration compared to other nucleosides [81]. Uridine is involved in several important biological pathways, such as the posttranslational modification of proteins via O-glycosylation, nucleic acid synthesis, and glycogen synthesis [82,83,84]. Uridine has also been reported to affect circadian rhythm, impacting several physiological processes, including sleep–wake cycles, body temperature, and metabolism [85]. The disruption of circadian rhythm resulting from the dysregulation of uridine can contribute to the progression of neurodegenerative diseases [86]. Moreover, uridine also plays a role in the formation of cell membranes and neuronal synapses, promotes the rejuvenation of aged stem cells and regeneration of various tissues, and thereby exerts an anti-aging effect. That is why an optimal level of uridine is needed to maintain the cellular processes and functions properly. Our result shows a significant decrease in uridine in NT1 patients compared to the control. IPA showed the association of uridine in activating pathways such as DNA damage, the development of cancer, and the synthesis of ROS.

A limitation of the study is the absence of standardized sleep questionnaires and objective sleep assessments in the control group, although all were clinically screened and reported no sleep complaints or relevant health issues.

## 5. Conclusions

In this study, serum metabolic profiling was conducted on 11 NT1 patients and 11 healthy controls, revealing that 453 metabolites were differentially abundant in NT1 out of a total of 1,491 metabolites. IPA analysis identified key biological pathways associated with the differentially abundant metabolites (DAMs), including inflammatory response, generation of reactive oxygen species, neuronal cell death, DNA damage, cancer, and immune cell response, all of which were predicted to be activated. Conversely, the activation of central nervous system cells and carbohydrate metabolism were predicted to be inhibited in NT1 patients.

Upregulated metabolites, such as linoleic acid and uric acid, were found to be involved in inflammatory responses and metabolic disorders. These findings are consistent with previous studies on the inflammation observed in metabolic diseases, such as type 2 diabetes, where elevated linoleic acid and uric acid levels were also reported. Among the downregulated metabolites, arginine, epinephrine, and uridine have been previously shown to influence the sleep–wake cycle and are associated with neurodegenerative diseases such as Alzheimer’s and Parkinson’s disease. Moreover, other validated upregulated differentially abundant metabolites, such as p-cresol sulfate, taurine, inosine, and malic acid, and downregulated metabolites, such as colchicine and corticosterone, may also be considered potential markers for NT1. However, due to the limited sample size of our current study, future studies with larger cohorts are necessary to further validate and establish these findings and proposed biomarkers.

Several amino acid metabolites were identified and found to be associated with important biological processes. Further studies are necessary to find the exact enantiomeric conformation of those amino acid metabolites and their roles in the development and progression of NT1.

## Figures and Tables

**Table 1:** Clinical information of the subjects.

Information	NT1	Control
Number of subjects	11	11
Age (years)	19–71	28–73
Sex (Male/Female)	7/4	7/4
Presence of HLA-DQB1*0602 allele	10	2
Cerebrospinal fluid hypocretin level (pg/mL)	0–119.6	Not available
Body Mass Index	20.2–28.6	Not available

**Table 2:** Main polysomnographic features of NT1 patients.

Parameter	Median (Interquartile Range)
Time in bed, min	445.0 (399.0–500.0)
Total Sleep Time, min	407.5 (348.0–461.0)
Sleep Onset Latency, min	6.0 (4.5–10.0)
First REM Latency, min	2.5 (1.0–56.5)
Number of Stage Shifts/Hour	12.6 (11.9–14.7)
Parameter	Median (Interquartile Range)
Number of Awakenings/hour	3.3 (1.9–4.7)
Sleep Efficiency, %	89.6 (82.1–95.3)
Sleep Stage N1, %	3.4 (1.7–7.0)
Sleep Stage N2, %	47.3 (45.6–53.8)
Sleep Stage N3, %	17.9 (14.9–23.2)
Sleep Stage R, %	28.2 (25.5–28.7)

## Data Availability

The raw files are available on Glycopost with accession number GPST000561.

## Supplementary Materials

The following supporting information can be downloaded at: https://www.mdpi.com/article/10.3390/metabo15060382/s1, Figure S1: Box plots of validated upregulated differentially abundant metabolites show the changes in the expression of the metabolites in NT1 compared to controls: (A–D) the box plots of p-cresol sulfate, taurine, ionize, and DL-malic acid from untargeted analysis, and (E–H) the box plots of the same metabolites from targeted PRM analysis; Figure S2: Box plots of validated downregulated differentially abundant metabolites show the changes in the expression of the metabolites in NT1 compared to controls: (A–E) the box plots of uridine, epinephrine, colchicine, corticosterone, and DL-arginine from untargeted analysis, and (E–H) the box plots of the same metabolites from targeted PRM analysis; Figure S3: Disease network from ingenuity pathway analysis shows several metabolites are involved in activating/inhibiting pathways, including cell-to-cell signaling and interaction, free radical scavenging, and nervous system function. The network also shows that some metabolites play a role in different functions (Fx), and some were previously reported as potential biomarkers (BMs); Figure S4: Disease network from ingenuity pathway analysis shows several metabolites are involved in some diseases and functions, including inflammatory disease, inflammatory response, organismal injury, and abnormalities. The network also shows that some of the metabolites implicated nervous system functions (Fx), leading to neurodegenerative conditions; Table S1: List of differentially abundant metabolites, average relative abundances of metabolites in control and NT1 samples, *p*-value, fold change, and log2 value of fold change; Table S2: List of differentially abundant metabolites validated by parallel reaction monitoring (PRM) analysis, their *m/z* value, transition ion fragments, fold change, and Log2 of FC of full scan and PRM experiments (Table annexed as an Excel file).

## Abbreviations

The following abbreviations are used in this manuscript:

NT1	Narcolepsy type 1
DCM	Dichloromethane
HLA	Human leukocyte antigen
CSF	Cerebrospinal fluid
MPA	Mobile phase A
MPB	Mobile phase B
HCD	High-energy collision dissociation
IPA	Ingenuity pathway analysis
HMDB	Human Metabolome Database
KEGG	Kyoto Encyclopedia of Genes and Genomes
PRM	Parallel reaction monitoring
PCA	Principal component analysis
ROS	Reactive oxygen species
GABA	Gamma-amino-butyric acid
DAMs	Differentially abundant metabolites
ROC	Receiver Operating Characteristics
FC	Fold change
AUC	Area under the curve
EDS	Excessive daytime sleepiness
REM	Rapid eye movement
NO	Nitric oxide
CNS	Central nervous system
