# Supplementary material for: Alteration of Metabolic Profile in Patients with Narcolepsy Type 1
Source: Metabolites. 2025 Jun 9;15(6):382. doi: 10.3390/metabo15060382 (PMC12195073; doi:10.3390/metabo15060382)
Supplement: Supplementary file 1 [file metabolites-15-00382-s001.zip › metabolites-3667769-supplementary.pdf]

[SUPPLEMENTARY INFORMATION]

Alteration of Metabolic Profile in Patients with Narcolepsy Type 1

Md Abdul Hakim[1‡], Waziha Purba[1‡], Akeem Sanni[1], Md Mostofa Al Amin Bhuiyan[1], Farid Talih[2], Giuseppe Lanza[3,4], Firas Kobeissy[5,6], Giuseppe Plazzi[7,8], Fabio Pizza[7,9], Raffaele Ferri[3], Yehia Mechref[1*]

[1]Chemistry and Biochemistry Department, Texas Tech University, Lubbock, Texas, USA

[2]Department of Psychiatry, Faculty of Medicine, American University of Beirut, Beirut, Lebanon

[3]Sleep Research Centre, Department of Neurology IC, Oasi Research Institute-IRCCS, Troina, Italy

[4]Department of Surgery and Medical-Surgical Specialties, University of Catania, Catania, Italy

[5]Department of Biochemistry and Molecular Genetics, Faculty of Medicine, American University of Beirut, Lebanon

[6]Department of Neurobiology, Center for Neurotrauma, Multiomics and Biomarkers (CNMB), Neuroscience Institute, Morehouse School of Medicine (MSM), Atlanta, GA, USA

[7]IRCCS, Instituto delle Scienze Neurologiche di Bologna, Bologna, Italy

[8]Department of Biomedical, Metabolic and Neural Sciences, University of Modena and Reggio Emilia, Modena, Italy

[9]Department of Biomedical and Neuromotor Sciences (DIBINEM), Alma Mater Studiorum, University of Bologna, Bologna, Italy

[‡] Authors contributed equally to this work

*Correspondence: Yehia Mechref, Ph.D.

Department of Chemistry and Biochemistry,

Texas Tech University

Lubbock, TX 79409-1061, USA.

Email: yehia.mechref@ttu.edu

Tel: 806-834-8246

Table of Contents

Supplementary Tables:

Supplementary Table S1. List of differentially abundant metabolites, average relative abundances of metabolites in control and NT1 samples, *p*-value, fold change, and log2 value of fold change.

Supplementary Table S2. List of differentially abundant metabolites validated by parallel reaction monitoring (PRM) analysis, their *m/z* value, transition ion fragments, fold change, and Log2 of FC of full scan and PRM experiment.

Supplementary Figures:

Supplementary Figure S1. Box plots of validated upregulated differentially abundant metabolites show the changes in the abundance of the metabolites in NT1 compared to controls; (A-D) the box plots of p-cresol sulfate, taurine, ionize, and DL-malic acid from untargeted analysis, (E-H) the box plots of the same metabolites from targeted PRM analysis.

Supplementary Figure S2. Box plots of validated downregulated differentially abundant metabolites show the changes in the abundance of the metabolites in NT1 compared to controls; (A-E) the box plots of uridine, epinephrine, colchicine, corticosterone, and DL-arginine from untargeted analysis, and (E-H) the box plots of the same metabolites from targeted PRM analysis.

Supplementary Figure S3. Disease network from ingenuity pathway analysis shows several metabolites are involved in activating/inhibiting pathways, including cell-to-cell signaling and interaction, free radical scavenging, and nervous system function. The network also shows that some metabolites play a role in different functions (Fx), and some were previously reported as potential biomarkers (BM).

Supplementary Figure S4. Disease network from ingenuity pathway analysis shows several metabolites are involved in some diseases and functions, including inflammatory disease, inflammatory response, organismal injury, and abnormalities. The network also shows that some of the metabolites implicated nervous system functions (Fx) leading to neurodegenerative conditions

Supplementary Table S1. List of differentially abundant metabolites, average relative abundances of metabolites in control and NT1 samples, *p*-value, fold change, and log2 value of fold change.

<u>Table annexed as an Excel file.</u>

Supplementary Table S2. List of differentially abundant metabolites validated by parallel reaction monitoring (PRM) analysis, their *m/z* value, transition ion fragments, fold change, and Log2 of FC of full scan and PRM experiment.

**Metabolites**	***m/z***	**Transition ion fragments**	**FC (Full scan)**	**Log2FC (Full scan)**	**FC (PRM)**	**Log2FC (PRM)**
Chlorotrifluoromethane	102.9566	87.9254,58.9588,65.6815	0.002	-8.80	0.92	-0.12
3-Indoxyl sulphate	212.0019	79.9574,92.0506,132.0455	0.002	-8.65	0.92	-0.12
Uridine	243.0616	140.0353,153.0305,183.0411	0.004	-7.81	0.55	-0.86
Prostaglandin D1	353.2328	177.0923,163.1129,60.7013	0.01	-7.34	0.89	-0.16
Cortolone-3-glucuronide	541.2650	75.0087,85.0296,113.0244	0.01	-6.50	0.76	-0.40
Cholic acid	409.2959	377.2669,289.1041,227.0594	0.01	-6.40	0.55	-0.85
L-(-)-3-Phenyllactic acid	165.0553	119.0502,72.9931,103.0554	0.02	-6.04	0.67	-0.58
1,7-Dimethyluric acid	195.0519	158.8467,160.8421,161.3769	0.02	-5.49	0.83	-0.27
5-Acetylamino-6-formylamino-3-methyluracil	225.0611	150.0173,168.0279,181.0595	0.02	-5.41	0.77	-0.37
Epinephrine	182.0818	114.0926,136.0768,138.0922	0.05	-4.46	0.81	-0.30
(R)-Malate	135.0299	65.0387,91.0543,107.0493	0.08	-3.67	0.77	-0.37
Colchicine	400.1767	216.6131,314.1794,383.2163	0.22	-2.21	0.80	-0.32
Corticosterone	391.2122	69.0345,83.0503,109.0659	0.25	-2.00	0.76	-0.39
1-Methylpyrrolinium	84.0815	57.0702,65.0389,67.0544	0.39	-1.35	0.50	-1.00
1-Methyladenosine	280.1041	94.9251,152.8836,92.928	0.47	-1.10	0.77	-0.38
N6-Acetyl-L-lysine	187.1080	143.119,74.0248,126.0925	0.51	-0.98	0.60	-0.75
2_5-Dioxopiperazine	115.0509	61.0399,76.6055,98.0238	0.52	-0.95	0.58	-0.78
3-Methylsulfolene	133.0324	61.011,75.0265,87.0264	0.57	-0.81	0.32	-1.63
Cer(d18:0/16:0)	540.5359	146.9819,256.2637,311.2948	0.59	-0.77	0.83	-0.26
2,5-di-tert-Butylhydroquinone	221.1542	97.9309,118.899,129.1782	0.61	-0.71	0.28	-1.82
DL-Arginine	173.1035	116.0718,129.1034,73.0408	0.66	-0.59	0.84	-0.25
trans-3-Indoleacrylic acid	186.0550	96.2806,117.0558,142.0661	0.66	-0.59	0.81	-0.30
N-stearoyl valine	384.3479	83.0856,69.0701,97.1013	0.74	-0.44	0.63	-0.66
Guaifenesin	197.0813	111.0451,135.0815,153.092	0.77	-0.37	0.67	-0.59
7-Hydroxycoumarine	163.0396	107.0492,135.0806,108.0526	0.79	-0.33	0.56	-0.84
Phthalic anhydride	149.0240	65.0388,98.0366,121.0286	0.84	-0.25	0.58	-0.79
20alpha-Hydroxy-4-pregnen-3-one	317.2482	105.0701,141.0703,257.2259	1.71	0.78	1.54	0.62
5-Hydroxyisourate	183.0156	97.0043,112.9993,140.0101	4.19	2.07	1.18	0.24
Monomethylsulfate	110.9755	79.9573,66.9945,95.9523	7.33	2.87	1.06	0.08
DL-Malic acid	133.0140	71.0138,115.0036,72.9931	7.85	2.97	1.07	0.09
4-Hydroxycinnamoylmethane	163.0759	50.8855,77.4318,121.065	11.73	3.55	1.06	0.09
2-Furoylglycine	170.0460	109.3599,115.0544,142.0654	12.52	3.65	1.07	0.10
3-(Decanoyloxy)-2-hydroxypropyl tridecanoate	441.3583	140.0117,196.0381,215.1653	31.48	4.98	1.34	0.43
3-Hydroxybenzoic acid	137.0241	53.8757,93.0345,96.4124	32.94	5.04	1.17	0.22
2-Hydroxycaproic acid	131.0711	85.0658,69.0345,113.0605	33.09	5.05	1.05	0.07
Diphenylmethane	169.1024	95.0493,96.0526,122.0319	45.30	5.50	1.01	0.02
5-L-Glutamyl-taurine	253.0495	124.0074,191.0498,106.9808	46.02	5.52	1.09	0.13

**Metabolites**	***m/z***	**Transition ion fragments**	**FC (Full scan)**	**Log2FC (Full scan)**	**FC (PRM)**	**Log2FC (PRM)**
4-Methylphenol	107.0500	95.05,63.9379,77.0395	47.30	5.56	4.87	2.28
2-Hydroxy2-methylbutyricacid	117.0555	71.0502,61.7974,55.0186	50.19	5.65	1.22	0.29
3-Ureidoisobutyrate	145.0616	102.056,84.0454,74.0247	52.76	5.72	1.18	0.24
12-Hydroxydodecanoic acid	215.1649	99.6141,135.0456,169.1599	100.19	6.65	1.50	0.58
10-hydroxydecanoicacid	187.1337	79.9574,80.9652,107.0502	180.31	7.49	1.72	0.79
Tetrahydroaldosterone-3-glucuronide	539.2500	75.0087,187.0069,333.2074	221.08	7.79	1.14	0.19
Inosine	267.0730	135.0314,92.0256,177.0419	429.74	8.75	1.18	0.23
L-Tyrosinemethylester	194.0820	151.1128,107.0502,135.0816	1245.96	10.28	1.44	0.53
CMPF	239.0922	135.0816,151.1128,195.1027	1257.48	10.30	1.08	0.12
Taurine	124.0071	79.9574,106.9807,64.9703	1315.48	10.36	1.21	0.28
p-Cresolsulfate	187.0068	79.9574,80.9652,107.0502	2601.71	11.35	1.73	0.79
N-Phenylacetylglutamine	265.1193	84.0445,91.0544,130.0501	4688.61	12.19	1.03	0.05

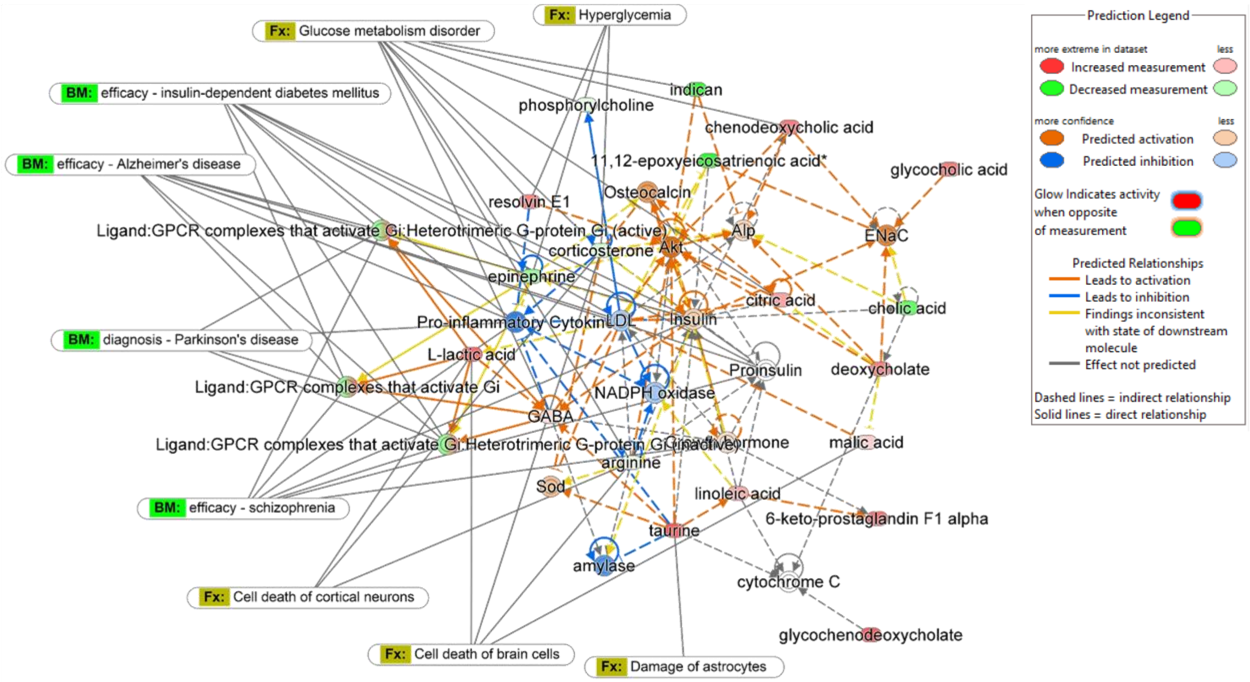

Supplementary Figure S1. Disease network from ingenuity pathway analysis shows several metabolites are involved in activating/inhibiting pathways, including cell-to-cell signaling and interaction, free radical scavenging, and nervous system function. The network also shows that some metabolites play a role in different functions (Fx), and some were previously reported as potential biomarkers (BM).

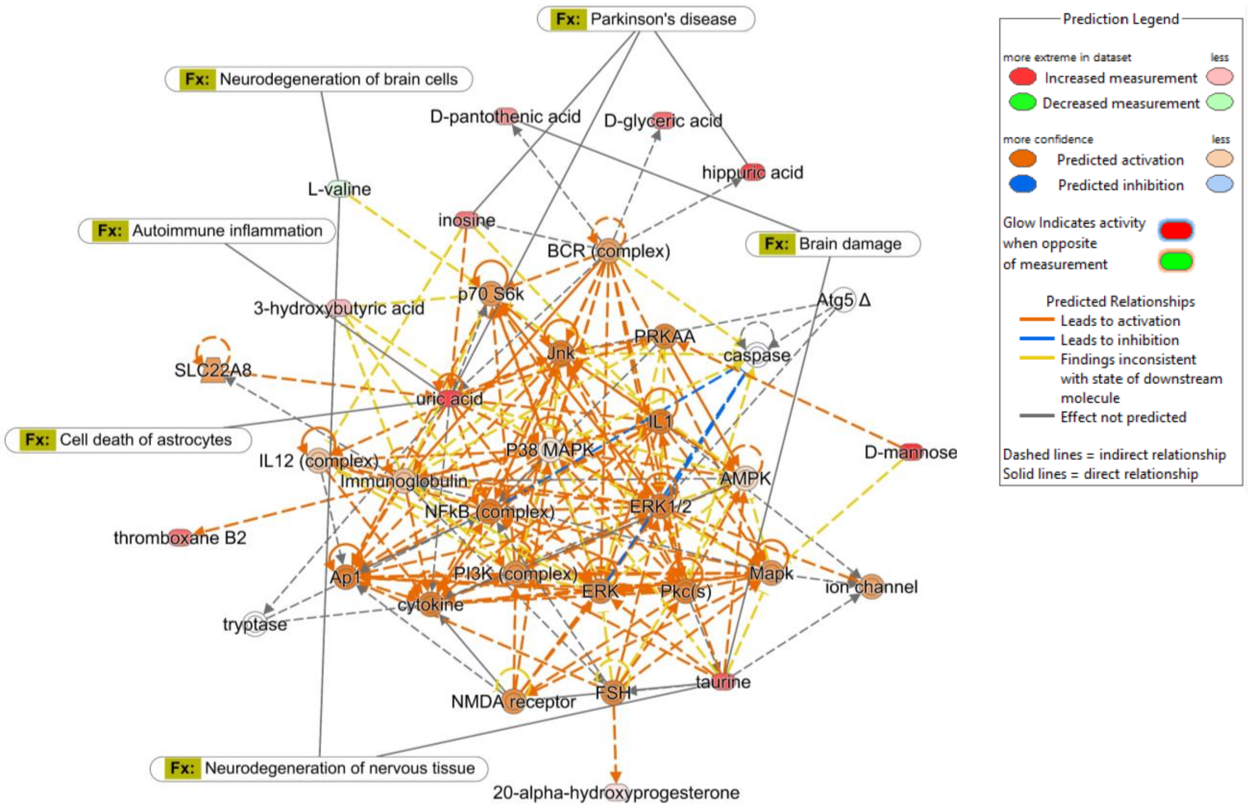

Supplementary Figure S2. Disease network from ingenuity pathway analysis shows several metabolites are involved in some diseases and functions, including inflammatory disease, inflammatory response, organismal injury, and abnormalities. The network also shows that some of the metabolites implicated nervous system functions (Fx) leading to neurodegenerative conditions

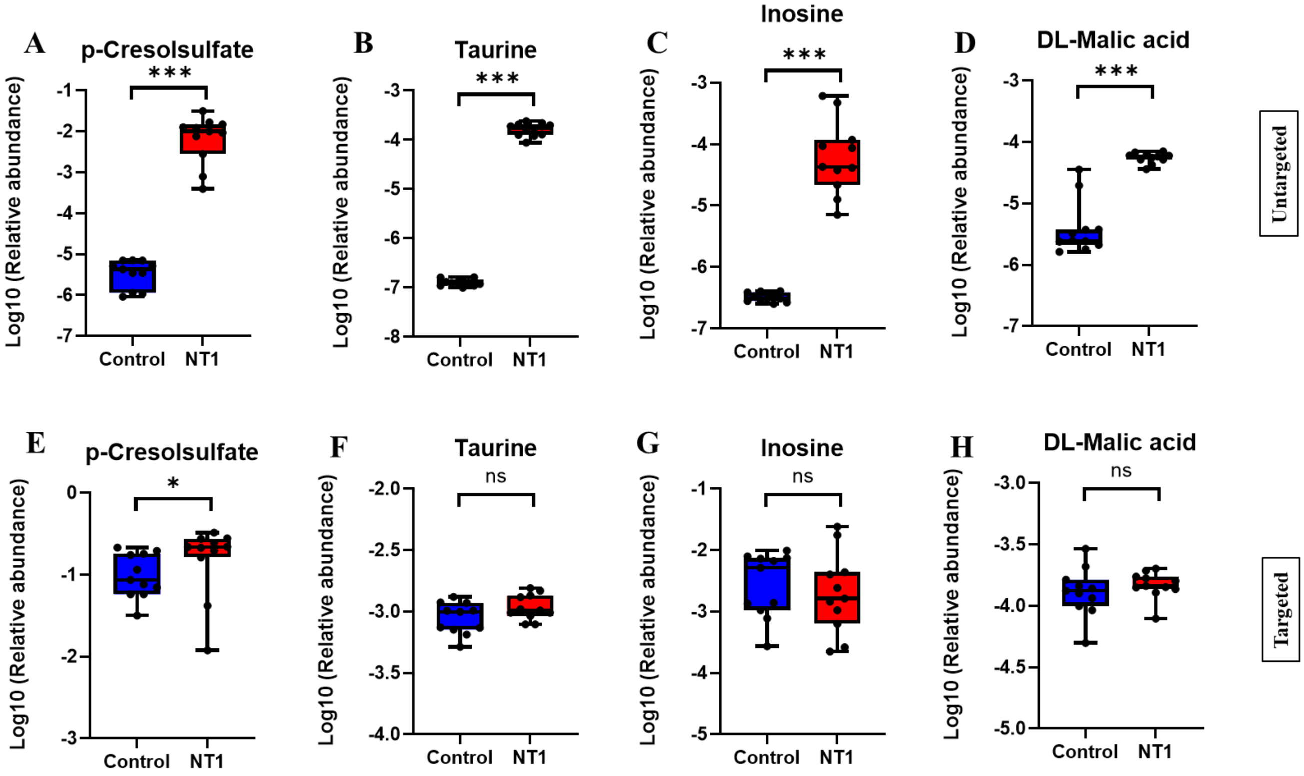

Supplementary Figure S3. Box plots of validated upregulated differentially abundant metabolites show the changes in the abundance of the metabolites in NT1 compared to controls; (A-D) the box plots of p-cresol sulfate, taurine, ionize, and DL-malic acid from untargeted analysis, (E-H) the box plots of the same metabolites from targeted PRM analysis.

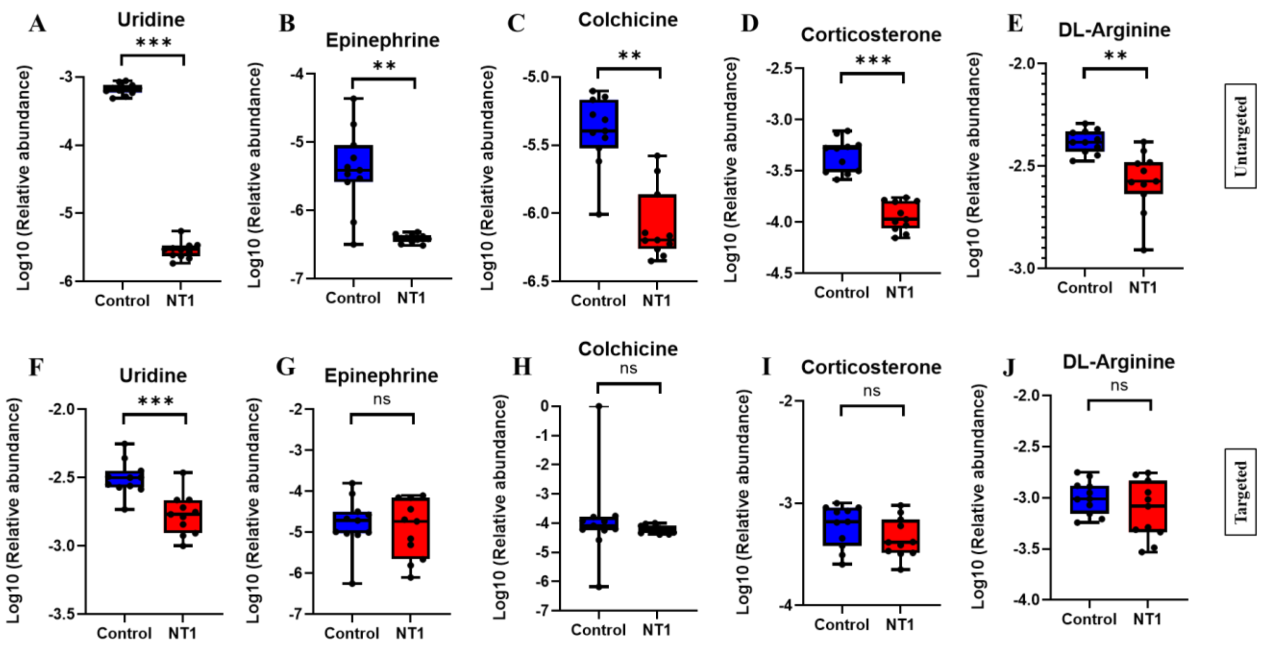

Supplementary Figure S4. Box plots of validated downregulated differentially abundant metabolites show the changes in the abundance of the metabolites in NT1 compared to controls; (A-E) the box plots of uridine, epinephrine, colchicine, corticosterone, and DL-arginine from untargeted analysis, and (E-H) the box plots of the same metabolites from targeted PRM analysis.

Name	Average (Control)	Average (NT1)	p-value	Fold Change (F(	Log2FC
Chlorotrifluorome	0.1±0.01	0.0003±0.00007	0.00001	0.0022	-8.8
11,12-Epoxy-(5Z	0.1±0.09	0.0003±0.0003	0.00001	0.0023	-8.73
4,4'-[1,5-Pentane	0.006±0.001	0.00002±0.000002	0.00001	0.0025	-8.66
3-Indoxyl sulpha	0.1±0.09	0.0004±0.00009	0.00001	0.0025	-8.65
D-(-)-Glutamine	0.06±0.01	0.0002±0.00003	0.00001	0.0029	-8.41
1-(2-methoxy-13	0.02±0.01	0.00008±0.00001	0.00001	0.0035	-8.14
Fademorf	0.01±0.003	0.00006±0.00001	0.00001	0.004	-7.95
Methyl 4-(9-meth	0.006±0.001	0.00002±0.000002	0.00001	0.0042	-7.91
Uridine	0.07±0.01	0.0003±0.0001	0.00001	0.0044	-7.81
L-Threonicacid	0.05±0.01	0.0002±0.0001	0.00001	0.0046	-7.77
2-Phenoxypropa	0.02±0.02	0.00007±0.00003	0.00001	0.0046	-7.77
N-(Chloroacetyl)	0.01±0.01	0.00006±0.000009	0.00001	0.0046	-7.76
8-iso Prostaglan	0.03±0.01	0.0001±0.00003	0.00001	0.0047	-7.74
N,N-Bis(2-chloro	0.01±0.002	0.00006±0.000005	0.00001	0.005	-7.65
1-(2-methoxy-eic	0.07±0.07	0.0004±0.0001	0.00001	0.0057	-7.46
MFCD00059603	0.003±0.001	0.00002±0.000002	0.00001	0.0057	-7.46
Prostaglandin D1	0.04±0.02	0.0002±0.00007	0.00001	0.0062	-7.34
L(+)-Citrulline;2-/	0.02±0.004	0.0001±0.00001	0.00001	0.0062	-7.33
7(S),17(S)-Dihyd	0.02±0.005	0.0001±0.00002	0.00001	0.0071	-7.13
(±)8-HEPE	0.01±0.008	0.00008±0.000009	0.00001	0.0079	-6.99
3-[N-(1-Carboxy-	0.003±0.003	0.00003±0.000003	0.00001	0.0082	-6.92
1,3,7-Trimethyl-8	0.02±0.01	0.0002±0.00005	0.00001	0.0084	-6.9
N-{[(1S,2S,10R,1	0.01±0.003	0.0001±0.00002	0.00001	0.0084	-6.9
LMST01010238	0.007±0.003	0.00006±0.000007	0.00001	0.0094	-6.73
PHHdiA-PE	0.02±0.004	0.0002±0.00006	0.00001	0.011	-6.52
Cortolone-3-gluc	0.02±0.005	0.0002±0.00007	0.00001	0.011	-6.5
Cholic acid	0.01±0.02	0.0002±0.00002	0.00001	0.012	-6.4
Azelaic acid	0.006±0.003	0.00008±0.00003	0.00001	0.012	-6.39
Naphthalen-2-an	0.002±0.001	0.00002±0.000003	0.00001	0.013	-6.26
4-[2-(2,3-Dihydro	0.01±0.006	0.0002±0.00002	0.00001	0.014	-6.16
3b,16a-Dihydrox	0.01±0.01	0.0002±0.00002	0.00001	0.014	-6.15
L-(-)-3-Phenyllac	0.006±0.006	0.0001±0.00004	0.00001	0.015	-6.04
2-Hydroxy-2,2-bi	0.02±0.008	0.0004±0.00007	0.00001	0.015	-6.04
16,17-Dihydroxy	0.007±0.005	0.0001±0.00001	0.00001	0.018	-5.8
N-(4,5-Dimethox	0.005±0.006	0.0001±0.00001	0.00001	0.020	-5.66
5-Acetylamino-6-	0.007±0.003	0.0002±0.0001	0.00001	0.024	-5.41
1-(Decanoyloxy)	0.008±0.006	0.0002±0.00007	0.00001	0.025	-5.35
Deoxycholicacid	0.003±0.003	0.00009±0.000009	0.00001	0.026	-5.24
CFM-1571	0.02±0.008	0.0007±0.0002	0.00001	0.027	-5.22
UNII:5D67SBH6	0.004±0.002	0.0001±0.00003	0.00001	0.027	-5.19
(Diethoxymethox	0.002±0.0009	0.00006±0.00002	0.00001	0.034	-4.87
Guaiacolsulfonat	0.006±0.007	0.0002±0.0001	0.00001	0.037	-4.76
2-(2-Hydroxyethy	0.005±0.009	0.0002±0.00003	0.00001	0.039	-4.7
S-glutathionyl-L-	0.001±0.0006	0.00005±0.000005	0.00001	0.039	-4.66
methyl 9,12-dihy	0.008±0.003	0.0003±0.0001	0.00001	0.044	-4.5
3,8,9-trihydroxy-	0.006±0.003	0.0004±0.00007	0.00001	0.056	-4.17
Prostaglandin K2	0.002±0.0007	0.0001±0.00001	0.00001	0.061	-4.05
(R)-Malate	0.006±0.002	0.0005±0.00008	0.00001	0.079	-3.67

Ethyl {2-[(2E)-5-(	0.007±0.004	0.0006±0.0001	0.00001	0.086	-3.54
9-Oxononanoica	0.002±0.001	0.0002±0.00009	0.00001	0.093	-3.42
2,3,4,5-tetrachlo	0.006±0.0008	0.0005±0.0007	0.00001	0.095	-3.4
5-Nitro-2-thiophe	0.1±0.06	0.02±0.01	0.00001	0.12	-3.09
1,2-Dichloro-3,3-	0.08±0.02	0.01±0.009	0.00001	0.15	-2.71
5,6-undecadien-8	0.02±0.006	0.003±0.003	0.00001	0.16	-2.67
Dioctyldimethyla	0.002±0.0005	0.0005±0.0005	0.00001	0.20	-2.34
acetyl triethyl citr	0.004±0.0008	0.001±0.0005	0.00001	0.24	-2.04
Corticosterone	0.05±0.02	0.01±0.004	0.00001	0.25	-2
His-His	0.006±0.002	0.002±0.0007	0.00001	0.27	-1.9
(2E)-N-[(3S,7S,1	0.01±0.005	0.003±0.0006	0.00001	0.28	-1.84
(1S_4S)-4-Hydro	0.01±0.003	0.003±0.0003	0.00001	0.31	-1.7
Bacillamidin A	0.0006±0.0002	0.0003±0.00008	0.00001	0.40	-1.32
Valine	0.7±0.1	0.3±0.1	0.00001	0.45	-1.15
1-Methyladenosi	0.006±0.001	0.003±0.0008	0.00001	0.47	-1.1
Cer(d32:0)	0.003±0.0003	0.001±0.0008	0.00001	0.50	-1
2-Amino-8-phosp	0.003±0.0005	0.001±0.0004	0.00001	0.52	-0.94
alpha-N-(3-hydro	0.001±0.0002	0.0006±0.0001	0.00001	0.52	-0.94
Cer(d18:0/16:0)	0.004±0.0003	0.002±0.001	0.00001	0.59	-0.77
4-Coumarylalcoh	0.004±0.0005	0.002±0.0004	0.00001	0.62	-0.7
3,4-Diphenyl-2,5	0.002±0.0003	0.002±0.0003	0.00001	0.66	-0.61
Isopropyl 4-[(2,4-	0.0005±0.0002	0.001±0.0001	0.00001	2.26	1.18
25FFJ4209Z	0.002±0.0006	0.006±0.001	0.00001	2.44	1.28
5-Hydroxyisoura	0.06±0.07	0.3±0.05	0.00001	4.19	2.07
Nimesulide	0.00003±0.000008	0.0001±0.0001	0.00001	4.34	2.12
N-Acetylalanine	0.002±0.0004	0.008±0.0009	0.00001	4.45	2.15
Pseudouridine	0.02±0.009	0.08±0.02	0.00001	4.70	2.23
Vinyl 6,6-dimethy	0.004±0.001	0.02±0.002	0.00001	4.81	2.27
2-Amino-3,5-dini	0.0005±0.0003	0.002±0.001	0.00001	4.82	2.27
20-beta-Dihydro	0.001±0.002	0.007±0.001	0.00001	4.99	2.32
UNII:OL56191M	0.0004±0.0002	0.002±0.0005	0.00001	5.57	2.48
Tensyuic acid B	0.0006±0.0005	0.003±0.0005	0.00001	5.65	2.5
N,6,7-Trimethyl-4	0.0003±0.00007	0.002±0.001	0.00001	6.68	2.74
3,3-Dimethylgluta	0.002±0.0001	0.01±0.002	0.00001	6.87	2.78
3-Acetoxystearic	0.0002±0.0002	0.002±0.0007	0.00001	6.89	2.78
(4-Formyl-5-hydr	0.00005±0.000007	0.0003±0.0008	0.00001	7.28	2.86
Monomethylsulfa	0.002±0.0006	0.01±0.001	0.00001	7.33	2.87
3-O-Butyryl-1,2-(	0.0007±0.0002	0.005±0.0005	0.00001	7.44	2.9
1-Chloro-4-[(4-m	0.00002±0.00001	0.0002±0.0001	0.00001	7.45	2.9
DL-Malic acid	0.0007±0.001	0.006±0.001	0.00001	7.85	2.97
Molybdenum(2+)	0.02±0.01	0.1±0.02	0.00001	7.93	2.99
Sulfosalicylic Aci	0.0002±0.0001	0.002±0.002	0.00001	8.17	3.03
DEEMM	0.003±0.002	0.02±0.004	0.00001	8.54	3.09
(-)-11-hydroxy-9,	0.0003±0.0001	0.003±0.0008	0.00001	8.61	3.11
[FAdioxo_hydrox	0.0006±0.0008	0.005±0.001	0.00001	9.03	3.17
16-Hydroxyhexa	0.002±0.003	0.02±0.006	0.00001	9.40	3.23
4-Hydroxybutyric	0.01±0.02	0.1±0.06	0.00001	9.82	3.3
YK6000000	0.0009±0.0008	0.009±0.0008	0.00001	9.83	3.3
Bicinchoninic aci	0.001±0.0007	0.01±0.01	0.00001	10.31	3.37

(±)10(11)-EpDPA	0.002±0.001	0.02±0.008	0.00001	10.83	3.44
D-Iditol	0.0003±0.0003	0.003±0.001	0.00001	12.12	3.6
26DEB00BBW	0.0009±0.0002	0.01±0.002	0.00001	12.43	3.64
2-Furoylglycine	0.00002±0.000004	0.0003±0.0002	0.00001	12.52	3.65
Carbofuran, 3OH	0.00005±0.00005	0.0007±0.0002	0.00001	13.55	3.76
Medronic Acid	0.008±0.006	0.1±0.03	0.00001	14.40	3.85
3-carboxy-4-met	0.003±0.005	0.05±0.02	0.00001	15.27	3.93
lixazinone	0.0008±0.0005	0.01±0.003	0.00001	15.60	3.96
(+/-)11(12)-EET	0.009±0.003	0.1±0.05	0.00001	15.67	3.97
N-Ribosylnicotina	0.00005±0.00001	0.0008±0.0009	0.00001	15.84	3.99
(3beta,5xi,6alpha	0.0005±0.0008	0.009±0.004	0.00001	16.06	4.01
MFCD00010184	0.0003±0.0002	0.005±0.002	0.00001	17.04	4.09
(+/-)9(10)-EpOM	0.0002±0.0002	0.005±0.004	0.00001	18.25	4.19
Trinonanoin	0.0001±0.0001	0.003±0.002	0.00001	19.82	4.31
Modavigil(R)	0.00004±0.000007	0.0008±0.002	0.00001	20.26	4.34
2-[3,8-Dihydroxy	0.0005±0.0003	0.01±0.001	0.00001	20.43	4.35
1-(Ethoxycarbon	0.0003±0.0001	0.006±0.0005	0.00001	20.54	4.36
N-Acetylornithine	0.0002±0.00007	0.005±0.002	0.00001	21.28	4.41
2-hydroxycaproic	0.0003±0.0003	0.007±0.004	0.00001	21.83	4.45
3-[(3-{(1R,2R,3R	0.0002±0.00008	0.005±0.005	0.00001	23.27	4.54
3beta-formyloxyr	0.00009±0.00004	0.002±0.0006	0.00001	23.45	4.55
Dodecanedioic a	0.0003±0.0004	0.007±0.002	0.00001	23.54	4.56
3-Hydroxybutyric	0.001±0.0005	0.03±0.03	0.00001	25.13	4.65
L-2-Amino-3-oxo	0.0002±0.00006	0.006±0.007	0.00001	26.31	4.72
2-Phenyl-4H-1,3	0.00002±0.000002	0.0005±0.002	0.00001	26.63	4.74
NP-008915	0.0001±0.00004	0.003±0.0004	0.00001	27.32	4.77
DL-Erythrono-1_	0.0007±0.0003	0.02±0.007	0.00001	28.99	4.86
Grandiflorone	0.000009±0.000003	0.0003±0.0007	0.00001	30.20	4.92
4-(2,3-DICHLOR	0.00002±0.000008	0.0007±0.0007	0.00001	31.06	4.96
3-(Decanoyloxy)-	0.00006±0.000006	0.002±0.0008	0.00001	31.48	4.98
Azelaicacid	0.0002±0.00006	0.005±0.002	0.00001	32.69	5.03
3-Hydroxybenzo	0.0001±0.00008	0.005±0.01	0.00001	32.94	5.04
2-Hydroxycaproi	0.0004±0.00005	0.01±0.009	0.00001	33.09	5.05
Ethyl 3,3-diethox	0.0001±0.00002	0.003±0.001	0.00001	33.64	5.07
Suberic acid	0.0002±0.00008	0.009±0.0009	0.00001	36.31	5.18
NP-017061	0.00005±0.000007	0.002±0.0009	0.00001	38.58	5.27
27-Nor-cholestar	0.00008±0.00001	0.003±0.001	0.00001	40.06	5.32
Gomisin D	0.00006±0.00001	0.002±0.001	0.00001	40.77	5.35
7-oxo-11E-Tetra	0.00006±0.00003	0.002±0.001	0.00001	41.08	5.36
Linoleic Acid	0.00006±0.00004	0.003±0.002	0.00001	41.10	5.36
Xeniasterol-b	0.00008±0.000009	0.003±0.002	0.00001	41.55	5.38
1,1'-[(E)-1,2-Ethe	0.0002±0.00007	0.009±0.003	0.00001	42.35	5.4
1a,1b-dihomo Pr	0.00008±0.00001	0.003±0.001	0.00001	43.24	5.43
Certonardosterol	0.00008±0.00001	0.004±0.001	0.00001	43.31	5.44
Isophthalic acid	0.00007±0.00003	0.003±0.0008	0.00001	43.93	5.46
PE(20:4(5Z,8Z,1	0.0003±0.0002	0.01±0.004	0.00001	44.11	5.46
Diphenylmethane	0.00006±0.000008	0.003±0.006	0.00001	45.30	5.5
6-[(2-Ethyl-5-oxo	0.00007±0.00004	0.003±0.0007	0.00001	45.78	5.52
Trinexapac-ethyl	0.00007±0.00006	0.003±0.0002	0.00001	45.89	5.52

5-L-Glutamyl-tau	0.0001±0.00003	0.005±0.001	0.00001	46.02	5.52
19(R)-hydroxy P	0.00002±0.00001	0.001±0.001	0.00001	47.55	5.57
2-methyl-hexade	0.00004±0.00001	0.002±0.0006	0.00001	48.00	5.58
5-[(1S,2R,4aR)-5	0.00007±0.000007	0.003±0.001	0.00001	49.03	5.62
Citric acid	0.002±0.001	0.1±0.03	0.00001	49.70	5.64
6alpha-Hydroxy-	0.0001±0.00001	0.005±0.001	0.00001	50.14	5.65
2-Hydroxy2-meth	0.002±0.003	0.1±0.1	0.00001	50.19	5.65
NP-003145	0.00005±0.00002	0.002±0.002	0.00001	50.25	5.65
2-(2,4-Dinitro-1H	0.0003±0.00009	0.01±0.003	0.00001	50.42	5.66
DG(13:0/20:4(5Z	0.00007±0.00001	0.003±0.003	0.00001	51.52	5.69
3-Ureidoisobutyr	0.00003±0.000009	0.002±0.001	0.00001	52.76	5.72
9-hydroxy-hexad	0.0001±0.0001	0.005±0.0007	0.00001	52.84	5.72
PCI-45227	0.00001±0.000002	0.0008±0.0005	0.00001	52.91	5.73
(2-((3,5-Di-t-buty	0.00008±0.00002	0.004±0.002	0.00001	53.22	5.73
2-[(1-Methoxy-3,	0.00007±0.000009	0.004±0.002	0.00001	53.45	5.74
NP-018716	0.0002±0.00006	0.008±0.001	0.00001	53.69	5.75
Anhydroecgonine	0.00005±0.00002	0.003±0.0009	0.00001	54.30	5.76
cholesteryl beta-	0.00007±0.00001	0.004±0.001	0.00001	54.40	5.77
Etretinate	0.00006±0.000008	0.003±0.002	0.00001	56.54	5.82
NP-016385	0.00005±0.00002	0.003±0.004	0.00001	58.06	5.86
2,13-Bis(2-methy	0.0001±0.00001	0.007±0.002	0.00001	58.09	5.86
Taxa-4(20)_11(1	0.00008±0.000009	0.005±0.002	0.00001	58.20	5.86
(-)-trans-Carveol	0.00001±0.000007	0.0009±0.001	0.00001	58.48	5.87
10-HDA	0.00009±0.0002	0.006±0.002	0.00001	60.29	5.91
Shikokianin B	0.0004±0.0007	0.02±0.01	0.00001	61.25	5.94
10,11-dihydro-20	0.0003±0.00008	0.02±0.004	0.00001	61.69	5.95
2-Methyl-2-propa	0.00002±0.000007	0.001±0.002	0.00001	62.33	5.96
3-(Sulfooxy)-L-ty	0.0002±0.0001	0.02±0.004	0.00001	63.03	5.98
6-{[1-(3,4-Dimeth	0.00006±0.00001	0.004±0.003	0.00001	63.66	5.99
NP-020353	0.0002±0.0001	0.01±0.002	0.00001	64.91	6.02
Ethyl-(5R,12S)-d	0.00005±0.000008	0.003±0.002	0.00001	66.77	6.06
NP-020078	0.00003±0.00001	0.002±0.002	0.00001	67.22	6.07
gamma-Tocotrie	0.00004±0.000005	0.003±0.003	0.00001	67.43	6.08
3-tert-Butyladipic	0.0002±0.0001	0.01±0.002	0.00001	69.54	6.12
2-CIDG	0.0001±0.00004	0.007±0.003	0.00001	70.68	6.14
MGMG(18:4(6Z,	0.0001±0.0002	0.008±0.005	0.00001	70.85	6.15
Furmecyclox	0.00005±0.000007	0.004±0.0005	0.00001	71.13	6.15
OHHdiA-PE	0.00006±0.00001	0.004±0.0007	0.00001	72.61	6.18
(R)-Pantolactone	0.007±0.0007	0.5±0.2	0.00001	73.03	6.19
1,18-Octadecane	0.00009±0.00001	0.006±0.002	0.00001	73.05	6.19
N~2~,N~6~-Bis[(	0.00002±0.000005	0.002±0.001	0.00001	79.77	6.32
Sarcoehrendin G	0.0001±0.00007	0.01±0.003	0.00001	82.12	6.36
5alpha-androsta	0.001±0.001	0.08±0.06	0.00001	84.82	6.41
N-[4-(3-Acetamid	0.00007±0.00004	0.006±0.008	0.00001	86.52	6.43
27-Norcholestan	0.00008±0.00001	0.007±0.004	0.00001	88.40	6.47
NP-003553	0.00009±0.00005	0.008±0.002	0.00001	89.95	6.49
13,14-dihydro-19	0.00004±0.000007	0.004±0.002	0.00001	92.88	6.54
trans-Petroselini	0.00005±0.000009	0.005±0.005	0.00001	94.11	6.56
4-(2-Ethylhexyl)	0.00009±0.00006	0.008±0.005	0.00001	95.35	6.58

[FAhydroxy(18:0	0.00006±0.00002	0.006±0.0009	0.00001	95.54	6.58
8-Chloro-1-naph	0.00002±0.00001	0.002±0.001	0.00001	95.95	6.58
3-hydroxy-tetrad	0.00005±0.00002	0.005±0.0006	0.00001	97.06	6.6
Limonoate	0.00003±0.000008	0.003±0.002	0.00001	98.73	6.63
1-[(Tetrahydro-2	0.00007±0.00001	0.007±0.0008	0.00001	98.94	6.63
D-(-)-Erythrose;L	0.0001±0.00002	0.01±0.008	0.00001	99.15	6.63
12-Hydroxydode	0.00004±0.00002	0.004±0.002	0.00001	100.19	6.65
3-carboxy-4-met	0.0004±0.00005	0.04±0.04	0.00001	102.53	6.68
Bis{2-[2-(methac	0.00002±0.000008	0.002±0.0005	0.00001	103.12	6.69
2,4,6-Trioxo-5-(1	0.00003±0.000006	0.003±0.002	0.00001	109.14	6.77
6-Deoxocastaste	0.0001±0.00001	0.01±0.004	0.00001	112.35	6.81
FAHFA(18:2/15-	0.00007±0.000009	0.008±0.006	0.00001	115.87	6.86
N-(2-{[3-(4-{2-[2-	0.00007±0.00002	0.009±0.002	0.00001	118.84	6.89
NP-022389	0.00003±0.000005	0.004±0.001	0.00001	122.44	6.94
Ancymidol	0.00002±0.000002	0.002±0.001	0.00001	123.28	6.95
Methylprednisolo	0.00006±0.000009	0.008±0.002	0.00001	126.96	6.99
delta10-13-Phyto	0.00005±0.00001	0.006±0.001	0.00001	127.54	6.99
diisooctyl sebaca	0.00007±0.00002	0.009±0.004	0.00001	129.11	7.01
Teasterone	0.0001±0.00002	0.02±0.005	0.00001	130.26	7.03
CH-13584	0.00003±0.00001	0.003±0.003	0.00001	130.36	7.03
D-(-)-Lyxose	0.00005±0.000009	0.007±0.002	0.00001	133.06	7.06
MFCD00067470	0.00004±0.00003	0.005±0.0007	0.00001	137.62	7.1
2-Butoxyethyl 4-(	0.00003±0.00001	0.005±0.0006	0.00001	137.84	7.11
N-(2,4,6-Trinitrop	0.0001±0.00002	0.02±0.006	0.00001	146.70	7.2
2-Hydroxyhippur	0.00001±0.000001	0.002±0.003	0.00001	149.00	7.22
β-Muricholic acid	0.0001±0.00005	0.02±0.05	0.00001	150.91	7.24
YQ2581000	0.0002±0.0002	0.04±0.03	0.00001	153.82	7.27
Linaprazan	0.00006±0.00001	0.009±0.002	0.00001	160.75	7.33
Pantothenic acid	0.00002±0.000003	0.003±0.0005	0.00001	166.27	7.38
(+/-)18-HEPE	0.00007±0.00001	0.01±0.003	0.00001	166.76	7.38
(+/-)19(20)-DiHD	0.00005±0.00001	0.009±0.003	0.00001	171.49	7.42
PE(O-18:1(9Z)/0	0.00005±0.000007	0.009±0.003	0.00001	179.48	7.49
10-hydroxydecan	0.00004±0.00004	0.007±0.003	0.00001	180.31	7.49
1,6-Bismaleimido	0.00002±0.000007	0.003±0.001	0.00001	182.00	7.51
Hexadecanedioic	0.00004±0.00002	0.007±0.0008	0.00001	186.38	7.54
15-oxo-11Z,13E-	0.00007±0.000008	0.01±0.004	0.00001	187.35	7.55
4S-hydroperoxy-	0.0003±0.00009	0.05±0.02	0.00001	188.58	7.56
(+/-)9-HpODE	0.00007±0.00005	0.01±0.004	0.00001	192.14	7.59
2-[(1-Methoxy-2,	0.00004±0.00001	0.008±0.002	0.00001	194.59	7.6
Androsterone glu	0.00003±0.00001	0.006±0.006	0.00001	198.32	7.63
D(+)-Phenyllactic	0.00004±0.00002	0.009±0.005	0.00001	204.24	7.67
Dibutyl itaconate	0.00003±0.00001	0.007±0.001	0.00001	204.61	7.68
laserpitin	0.00009±0.00001	0.02±0.03	0.00001	206.77	7.69
N,N'-(Iminodi-2,1	0.000004±0.0000005	0.0008±0.0002	0.00001	207.33	7.7
delta5-8-NeuroF	0.00008±0.00001	0.02±0.004	0.00001	208.13	7.7
Resolvin E1	0.00008±0.00001	0.02±0.005	0.00001	210.76	7.72
Tetrahydroaldost	0.00003±0.000008	0.007±0.002	0.00001	221.08	7.79
5-hydroxy-2,2,6,(	0.0001±0.00007	0.03±0.02	0.00001	223.30	7.8
3,4-Dihydroxybe	0.0002±0.00006	0.04±0.03	0.00001	230.75	7.85

1-(4-Butoxyphen	0.0002±0.0001	0.05±0.03	0.00001	235.22	7.88
3-Oxododecanoi	0.00003±0.000008	0.006±0.0008	0.00001	249.93	7.97
PS(19:0/0:0)	0.00008±0.00001	0.02±0.007	0.00001	251.60	7.98
Cer(d18:0/22:0(2	0.000007±0.0000008	0.002±0.0002	0.00001	252.47	7.98
MFCD18428366	0.00009±0.00001	0.02±0.02	0.00001	256.74	8
Deoxycholic Acid	0.0001±0.00002	0.03±0.02	0.00001	259.74	8.02
3-methyl-tetrade	0.00002±0.000007	0.005±0.0006	0.00001	262.27	8.03
14(S)-HDHA	0.00007±0.00001	0.02±0.009	0.00001	269.94	8.08
(2E)-3-(Acetoxyr	0.00003±0.00001	0.007±0.001	0.00001	277.02	8.11
LPE(16:0)	0.00006±0.000006	0.02±0.007	0.00001	282.57	8.14
Chenodeoxychol	0.00007±0.000009	0.02±0.02	0.00001	289.53	8.18
Tetradecanedioi	0.00004±0.00003	0.01±0.004	0.00001	317.75	8.31
Glycocholic acid	0.00003±0.00004	0.01±0.01	0.00001	326.03	8.35
(15Z)-9,12,13-Tr	0.00002±0.00001	0.008±0.002	0.00001	333.48	8.38
8-Isoprostagland	0.00004±0.00001	0.01±0.006	0.00001	344.59	8.43
3-Dehydroteaste	0.00009±0.00001	0.03±0.008	0.00001	356.14	8.48
D-Arabinonate	0.00004±0.00001	0.02±0.007	0.00001	388.42	8.6
Testosterone sul	0.0007±0.0004	0.3±0.2	0.00001	422.62	8.72
Inosine	0.00003±0.000005	0.01±0.02	0.00001	429.74	8.75
Hexyl fumarate	0.00002±0.000008	0.007±0.0006	0.00001	443.21	8.79
(Z)-7-(5-((1E,3E,	0.00006±0.000009	0.03±0.009	0.00001	468.99	8.87
Δ17-6-keto prost	0.0001±0.00003	0.06±0.01	0.00001	472.14	8.88
DL-4-Hydroxyph	0.00005±0.00003	0.02±0.007	0.00001	490.92	8.94
13,14-dihydro-15	0.00003±0.000009	0.01±0.002	0.00001	498.33	8.96
Glycoursodeoxy	0.00001±0.000006	0.006±0.005	0.00001	506.55	8.98
Thromboxane B2	0.00006±0.00001	0.03±0.006	0.00001	554.38	9.11
tenofovir exalide	0.0001±0.0001	0.09±0.02	0.00001	615.20	9.26
(2R)-2,3-Dihydro	0.00002±0.00001	0.01±0.004	0.00001	631.10	9.3
L-(+)-Lactic acid	0.002±0.002	1.1±0.3	0.00001	631.20	9.3
8,12-iso-iPF2α-V	0.00003±0.00001	0.02±0.009	0.00001	711.69	9.48
17(S)-HpDHA	0.00006±0.00002	0.05±0.01	0.00001	912.24	9.83
2-Carboxy-D-ara	0.00001±0.000004	0.01±0.005	0.00001	972.00	9.92
Glycochenodeox	0.00006±0.00001	0.06±0.06	0.00001	1003.53	9.97
Indole-3-lactic ac	0.00002±0.000006	0.02±0.007	0.00001	1067.46	10.06
Dibutyl ethylmalo	0.00001±0.000006	0.02±0.002	0.00001	1138.35	10.15
L-Tyrosinemethy	0.00003±0.000009	0.03±0.01	0.00001	1245.96	10.28
CMPF	0.0001±0.0001	0.2±0.3	0.00001	1257.48	10.3
Taurine	0.00001±0.000002	0.02±0.005	0.00001	1315.48	10.36
Ethylphosphonot	0.00001±0.000001	0.01±0.001	0.00001	1411.26	10.46
LPE(18:0)	0.0003±0.00006	0.4±0.09	0.00001	1542.97	10.59
D-(+)-Tryptophar	0.00002±0.000003	0.03±0.006	0.00001	1671.29	10.71
Telluroxomagnes	0.00001±0.000002	0.02±0.003	0.00001	1803.81	10.82
L-Threonic acid	0.00003±0.00001	0.05±0.02	0.00001	2017.33	10.98
p-cresolsulfatep	0.0004±0.0002	1.1±0.9	0.00001	2601.71	11.35
1-(2-methoxy-oc	0.0001±0.00006	0.4±0.09	0.00001	2951.98	11.53
Uric acid	0.0003±0.00008	1±0.5	0.00001	3045.09	11.57
Hippuric acid	0.00004±0.00001	0.2±0.2	0.00001	4050.81	11.98
N-Phenylacetylg	0.00001±0.000003	0.07±0.04	0.00001	4688.61	12.19
12-Epileukotrien	0.00004±0.00001	0.2±0.04	0.00001	4768.16	12.22

6-Chloro-6-deox	0.00002±0.00001	0.1±0.04	0.00001	5083.71	12.31
D-(+)-Mannose	0.0003±0.0001	1.7±0.4	0.00001	6761.29	12.72
Telluroxobarium	0.000009±0.000001	0.09±0.02	0.00001	10823.30	13.4
1,2-Benzisothiaz	0.001±0.0007	0.0003±0.0002	0.00003	0.18	-2.48
LMFA08020332	0.0007±0.0002	0.0002±0.00009	0.00003	0.23	-2.15
Nitrofurazone	0.07±0.01	0.03±0.008	0.00003	0.48	-1.05
N6-Acetyl-L-lysin	0.004±0.0008	0.002±0.0005	0.00003	0.51	-0.98
methadone-d9	0.02±0.04	0.1±0.01	0.00003	5.11	2.35
5-[8,10-Dihydrox	0.0004±0.0009	0.005±0.002	0.00003	13.32	3.74
4-Methylphenol	0.0003±0.0001	0.01±0.01	0.00003	47.30	5.56
(2S,3S)-3-Amino	0.01±0.003	0.005±0.002	0.00004	0.36	-1.47
Roridan C	0.002±0.0005	0.0008±0.0003	0.00004	0.44	-1.18
Subericacid	0.01±0.004	0.0002±0.0002	0.00005	0.021	-5.6
Amdoxovir	0.01±0.006	0.003±0.003	0.00005	0.22	-2.18
NP-006862	0.001±0.0006	0.0003±0.0002	0.00005	0.24	-2.04
6,15-diketo-13,1	0.0008±0.0003	0.0002±0.00009	0.00005	0.32	-1.63
Methyltrifluorosil	0.03±0.007	0.02±0.003	0.00005	0.54	-0.9
Ovalitenin A	0.03±0.003	0.02±0.003	0.00005	0.72	-0.46
Umespirone	0.01±0.007	0.0001±0.00001	0.00007	0.0097	-6.69
Carbamazepine	0.001±0.004	0.000002±0.0000005	0.00008	0.0016	-9.25
Diethyl 4-oxopim	0.03±0.008	0.009±0.005	0.00008	0.33	-1.59
3-[(3-Hydroxyhe	0.001±0.0002	0.0006±0.00009	0.00008	0.66	-0.61
Stieleriacine B1	0.0002±0.0001	0.00006±0.00004	0.00009	0.24	-2.04
Ethanol, 2,2â€²-[	0.0007±0.0003	0.0002±0.0001	0.00009	0.31	-1.71
DILAURYLMETH	0.003±0.0007	0.002±0.001	0.00009	0.51	-0.96
N,N-Dimethylani	0.1±0.02	0.07±0.01	0.00009	0.65	-0.63
2-Methoxy-5-sul	0.00007±0.00002	0.009±0.03	0.00009	120.52	6.91
(±)9(10)-EpOME	0.01±0.004	0.0007±0.0006	0.0001	0.068	-3.89
(5Z_9E_14Z)-(8	0.01±0.01	0.0001±0.00002	0.0002	0.0094	-6.73
glas#3	0.01±0.005	0.0004±0.0001	0.0002	0.036	-4.79
N-(1-Ethyl-3-pipe	0.006±0.007	0.0005±0.0009	0.0002	0.076	-3.72
Colchicine	0.0005±0.0002	0.0001±0.00007	0.0002	0.22	-2.21
3-{[2-{[(6-Aminoh	0.002±0.0008	0.0005±0.0002	0.0002	0.29	-1.81
12-{4-[4-(2-Pyrim	0.0004±0.0002	0.0001±0.00005	0.0002	0.32	-1.66
6-Amino-1,3-dim	0.0002±0.0001	0.00006±0.00001	0.0002	0.32	-1.65
PHA-793887	0.0008±0.0003	0.0003±0.0002	0.0002	0.37	-1.43
5-Hydroxyindole	0.004±0.001	0.002±0.0005	0.0002	0.48	-1.07
Methylarsonic ac	0.3±0.08	0.5±0.07	0.0002	1.58	0.66
Acetaminophen	0.00007±0.00002	0.007±0.02	0.0002	95.68	6.58
9_12-Dioxodode	0.01±0.003	0.009±0.002	0.0003	0.58	-0.79
LPS(21:0)	0.05±0.05	0.0001±0.00003	0.0004	0.0022	-8.86
N,N'-Bis{6-[(2-m	0.02±0.03	0.0003±0.0003	0.0004	0.012	-6.42
N~6~,N~6~-Dim	0.02±0.005	0.007±0.003	0.0004	0.43	-1.22
N-Methyldioctyla	0.005±0.001	0.003±0.001	0.0004	0.56	-0.84
[FAoxo(12:0)]12-	0.006±0.001	0.01±0.003	0.0004	1.89	0.92
7-methyl-6E-hex	0.009±0.004	0.02±0.004	0.0004	2.26	1.17
Solacetal A	0.00005±0.00001	0.001±0.003	0.0004	22.75	4.51
(3beta,5alpha,6a	0.0004±0.0002	0.0001±0.0001	0.0005	0.28	-1.82
TA-5707F	0.002±0.0006	0.0007±0.0006	0.0005	0.35	-1.51

Methanesulfonar	0.002±0.0005	0.0008±0.0003	0.0005	0.46	-1.14
Dimethyl phosph	0.03±0.006	0.02±0.006	0.0005	0.63	-0.67
Trimethyl(pheno:	0.7±0.09	0.4±0.2	0.0005	0.63	-0.67
Iron(2+) dihydrox	0.05±0.02	0.03±0.009	0.0005	0.63	-0.66
trans-3-Indoleac	2.7±0.6	1.8±0.3	0.0005	0.67	-0.59
N-stearoyl valine	0.009±0.001	0.006±0.0009	0.0005	0.74	-0.44
Tetraethylene gly	0.002±0.005	0.02±0.003	0.0005	9.23	3.21
Helicin	0.00008±0.00006	0.005±0.005	0.0005	56.90	5.83
4-Hydroxy nimes	0.00002±0.000002	0.0001±0.0001	0.0006	7.97	2.99
MFCD00083078	0.002±0.0008	0.0004±0.0005	0.0007	0.26	-1.93
Amiprilose	0.0006±0.0003	0.00008±0.00007	0.0008	0.13	-2.92
3-Methylsulfolen	0.5±0.1	0.3±0.1	0.0008	0.57	-0.81
1-Octanesulfony	0.1±0.02	0.1±0.02	0.0008	0.76	-0.4
3-(3-methylfuran	0.0001±0.00006	0.001±0.002	0.0008	10.62	3.41
1-Methyluric acid	0.0001±0.0003	0.002±0.002	0.0008	12.64	3.66
1-{2-[5-(3,4-Dime	0.006±0.003	0.0009±0.002	0.001	0.15	-2.72
N-[2-({(5R,6S)-5-	0.0007±0.0004	0.0002±0.00008	0.001	0.25	-1.98
Stearamidopropy	0.004±0.002	0.002±0.001	0.001	0.41	-1.29
DL-Arginine	0.4±0.05	0.3±0.08	0.001	0.66	-0.59
p-Cresol, 2,2'-me	0.002±0.0006	0.004±0.001	0.001	1.86	0.89
Suspensolide	0.004±0.001	0.008±0.003	0.001	1.86	0.89
(+/-)12(13)-DiHO	0.02±0.03	0.09±0.02	0.001	6.05	2.6
1,7-Dimethyluric	0.0009±0.001	0.00002±0.000003	0.002	0.022	-5.49
4-mesylphenylac	0.008±0.008	0.0003±0.00003	0.002	0.041	-4.63
Epinephrine	0.0009±0.001	0.00004±0.000005	0.002	0.045	-4.46
benzyl ?-D-gluco	0.004±0.003	0.0002±0.00006	0.002	0.055	-4.18
Metazosin	0.001±0.001	0.00007±0.00003	0.002	0.056	-4.17
Tyr-gly-gly	0.003±0.003	0.0007±0.0004	0.002	0.21	-2.28
2,2'-(1H-Imidazo	0.004±0.0004	0.001±0.002	0.002	0.26	-1.94
22-(1-Hydroxyeth	0.002±0.0007	0.0006±0.0003	0.002	0.34	-1.55
2-Oxo-delta3-4_5	0.03±0.01	0.01±0.01	0.002	0.39	-1.38
3-Hexadecyl-1,1	0.002±0.0008	0.0008±0.0005	0.002	0.40	-1.31
(2R,3R,4R)-2-An	0.2±0.08	0.09±0.05	0.002	0.41	-1.27
MFCD03094680	0.005±0.01	0.002±0.007	0.002	0.50	-1.01
35-aminobacteri	0.0006±0.0001	0.0003±0.0001	0.002	0.61	-0.7
4-Methyl-3-propo	0.3±0.05	0.2±0.08	0.002	0.63	-0.67
(9E)-N,N-Bis[2-(2	0.002±0.0003	0.001±0.0005	0.002	0.65	-0.63
2,4-Dichlorobenz	0.0002±0.00002	0.0001±0.00004	0.002	0.69	-0.54
(+/-)-panthenol tr	0.003±0.0003	0.002±0.002	0.002	0.71	-0.49
MFCD00042968	0.01±0.001	0.01±0.002	0.002	0.79	-0.34
3-Methoxy-4-hyd	0.01±0.003	0.01±0.004	0.002	0.80	-0.32
NP-003459	0.1±0.03	0.1±0.07	0.002	0.82	-0.28
2,3,4,5-tetranor-t	0.0009±0.001	0.002±0.0008	0.002	2.23	1.16
1-Deoxy-1-[(3,4-	0.003±0.001	0.02±0.007	0.002	5.71	2.51
gamma-Aminobu	0.002±0.004	0.01±0.007	0.002	5.75	2.52
Amixetrine	0.0009±0.0007	0.009±0.03	0.002	9.24	3.21
CAPROYL SARC	0.002±0.002	0.02±0.009	0.002	11.36	3.51
bencyclane	0.0006±0.0004	0.008±0.03	0.002	12.74	3.67
3,4,2',4',6'-Penta	0.0007±0.002	0.00003±0.00003	0.003	0.040	-4.64

Methyl [(2Z)-3-{4	0.01±0.01	0.0009±0.001	0.003	0.087	-3.53
Isophorone diam	0.0006±0.0006	0.0001±0.0002	0.003	0.18	-2.48
D-1-Aminopropa	0.0004±0.0002	0.0001±0.0001	0.003	0.29	-1.8
Ethyl 3,4,4-trime	0.001±0.0007	0.0004±0.0006	0.003	0.30	-1.75
1-Methylpyrrolini	0.02±0.008	0.007±0.005	0.003	0.39	-1.35
1-Butanol, 4-(but	0.004±0.001	0.002±0.0009	0.003	0.46	-1.13
Cetrimonium	0.02±0.005	0.01±0.007	0.003	0.48	-1.07
H-Arg-AMC	0.004±0.001	0.002±0.002	0.003	0.51	-0.98
NP-016596	0.0009±0.0002	0.0005±0.0002	0.003	0.60	-0.74
L-N2-(2-Carboxy	0.004±0.001	0.002±0.0005	0.003	0.60	-0.73
2,5-di-tert-Butylh	0.01±0.002	0.006±0.003	0.003	0.61	-0.71
3-Oxalomalate	0.003±0.0007	0.002±0.0004	0.003	0.63	-0.66
TY7350000	0.000007±0.00001	0.000005±0.000008	0.003	0.73	-0.44
Pebulate	0.002±0.0003	0.001±0.0003	0.003	0.74	-0.43
Tetranor-PGEM	0.01±0.001	0.009±0.002	0.003	0.77	-0.37
7-Hydroxycouma	0.003±0.0005	0.002±0.0006	0.003	0.79	-0.33
1,1,12,12-Dodec	0.0005±0.0003	0.002±0.001	0.003	2.89	1.53
2-Chloro-N-(2-ch	0.009±0.01	0.0008±0.001	0.004	0.090	-3.48
Buspirone	0.0004±0.0003	0.00008±0.00003	0.004	0.22	-2.2
4-Coumarylalcoh	0.02±0.008	0.005±0.007	0.004	0.30	-1.73
2-[4-(2,3,4-Trime	0.003±0.0005	0.002±0.0009	0.004	0.63	-0.68
Laurolactam	0.002±0.0004	0.001±0.0002	0.004	0.70	-0.51
Dosulepin	0.04±0.006	0.03±0.008	0.004	0.71	-0.5
Bis(3,5,5-trimeth	0.002±0.0005	0.002±0.0004	0.004	0.73	-0.45
3-Ethyl-4-oxo-3,4	0.05±0.008	0.04±0.01	0.004	0.73	-0.44
Methyl (5alpha,1	0.00006±0.000008	0.00005±0.000006	0.004	0.80	-0.32
Meperidine	0.0008±0.0001	0.003±0.007	0.004	3.49	1.8
Prenyl-L-cysteine	0.0003±0.0007	0.002±0.003	0.004	5.41	2.43
4-Hydroxycinnan	0.0007±0.00009	0.008±0.005	0.004	11.73	3.55
Prohexadione	0.00002±0.000004	0.0005±0.001	0.004	30.42	4.93
1-(2,2-Diethoxye	0.001±0.0005	0.0005±0.0003	0.005	0.39	-1.36
2_5-Dioxopipera	0.004±0.001	0.002±0.001	0.005	0.52	-0.95
(1-Hydroxy-4-oxi	0.008±0.002	0.004±0.003	0.005	0.57	-0.82
Dinitrosopentam	0.004±0.001	0.002±0.0008	0.005	0.65	-0.63
2-(6-Nitro-1H-be	0.01±0.002	0.008±0.004	0.005	0.71	-0.5
2-(1-Hydroxycyc	0.02±0.003	0.01±0.002	0.005	0.72	-0.47
Chromic acid	0.1±0.04	0.1±0.03	0.005	0.72	-0.47
Guaifenesin	0.02±0.003	0.02±0.007	0.005	0.77	-0.37
Phthalic anhydric	0.01±0.002	0.01±0.001	0.005	0.84	-0.25
MFCD08460923	0.04±0.01	0.05±0.009	0.005	1.49	0.58
Ethyl 4-nitropher	0.004±0.003	0.001±0.002	0.006	0.23	-2.15
Azapropazone	0.002±0.0007	0.001±0.0009	0.006	0.52	-0.93
NP-021018	0.02±0.003	0.01±0.004	0.006	0.68	-0.56
1-[Methyl(nitroso	0.005±0.001	0.003±0.0006	0.006	0.68	-0.55
1,3,5-Trinitro-2,4	0.00007±0.00002	0.00006±0.00002	0.006	0.82	-0.29
Chlorotrifluoroetl	0.02±0.004	0.02±0.006	0.006	1.43	0.51
hydroxyprocaine	0.00002±0.00001	0.00006±0.00005	0.006	3.09	1.63
LTB4 ethanol am	0.0003±0.0001	0.00008±0.00004	0.008	0.30	-1.74
N6-PALMITOYL	0.0003±0.0001	0.0001±0.00006	0.008	0.51	-0.98

(-)-alpha-Cedren	0.002±0.0006	0.0008±0.0003	0.008	0.52	-0.95
NP-021797	0.01±0.003	0.007±0.002	0.008	0.64	-0.64
Ageratriol	0.007±0.001	0.005±0.001	0.008	0.70	-0.52
1-(beta-D-Arabin	4.7±0.6	3.6±0.9	0.008	0.76	-0.4
Docosanamide	0.02±0.003	0.02±0.002	0.008	0.84	-0.24
20alpha-Hydroxy	0.006±0.003	0.01±0.004	0.008	1.71	0.78
Ipronidazole	0.002±0.004	0.007±0.004	0.008	4.06	2.02
1,4-Dihydroxy-5,	0.00003±0.00004	0.0007±0.001	0.008	21.69	4.44
4'-Methylthioprop	0.01±0.001	0.01±0.004	0.009	0.70	-0.51
3-O-(N-acetyl-be	0.03±0.007	0.02±0.008	0.01	0.68	-0.56
Cholinephosphat	0.02±0.002	0.01±0.008	0.01	0.75	-0.42
N,N-DIMETHYLF	0.002±0.0002	0.002±0.0003	0.01	0.79	-0.34
2-Hydroxy-2_4-p	0.003±0.001	0.004±0.002	0.01	1.79	0.84
